# Mechanistic insights into modified Danggui Buxue Decoction for diabetic retinopathy via integrative analysis

**DOI:** 10.3389/fendo.2025.1648831

**Published:** 2025-10-10

**Authors:** Lee Yam Poon, Li Mei Hsu, Lai Kwan Lam, Xiaoqing Huang, Pengli Xu, Qiuer Liang, Pengcheng Xie, Shuyu Yang

**Affiliations:** ^1^ Center of Integrated Chinese and Western Medicine, The First Affiliated Hospital of Xiamen University, School of Medicine, Xiamen University, Xiamen, Fujian, China; ^2^ School of Traditional Chinese Medicine, Jinan University, Guangzhou, China; ^3^ Department of Obstetrics and Gynaecology, Faculty of Medicine, The Chinese University of Hong Kong, Hong Kong, Hong Kong SAR, China; ^4^ Affiliated Dongguan People’s Hospital, Southern Medical University, Dongguan, China; ^5^ The Third Affiliated Hospital, Guangzhou University of Traditional Chinese Medicine, Guangzhou, China

**Keywords:** MDBD, beta-sitosterol, diabetic retinopathy, network pharmacology, molecular docking, Mendelian randomization, *in vitro* validation

## Abstract

**Objective:**

This study explores the therapeutic potential and mechanisms of Modified Danggui Buxue Decoction (MDBD) in diabetic retinopathy (DR) using network pharmacology, bioinformatics, machine learning, Mendelian randomization (MR), molecular docking, and *in vitro* experiments.

**Methods:**

A network pharmacology was constructed in order to screen core components and targets. Analysis of samples from the GEO database was performed for target and immune cell analysis, resulting in the identification of significantly differentially expressed core genes (SDECGs). A machine learning model was utilized to screen feature genes and construct nomogram. Preliminary validation was carried out using molecular docking, another GEO dataset, and MR. Subsequently, samples were clustered based on SDECGs expression and consensus clustering, followed by an analysis between clusters. SDECGs expression was scored and differences between clusters were analyzed. Finally, *in vitro* experiments were conducted on MMCs to assess the effects of beta-sitosterol, the primary active component of MDBD, and siRNA on DR-related biomarkers using CCK-8 assays, ELISA, western blotting and RT-qPCR.

**Results:**

This study identified the core components of MDBD, including quercetin, stigmasterol, beta-sitosterol, kaempferol, and 14 differentially expressed SDECGs between DR and control groups, with both positive and negative immune cell regulatory effects. Five feature genes (CCND1, ERBB2, INSR, TP53, SERPINE1) were identified and used to construct a predictive model. MR analysis revealed a causal link between elevated ERBB2 levels and increased DR risk (Odds Ratio [OR]=1.860, 95% CI: 1.247-2.774, *P* = 0.002) using the weighted median method. Beta-sitosterol displayed high binding affinity with CCND1, ERBB2, INSR, and SERPINE1. Cluster analysis categorized DR samples into four groups, with C1 showing low and C2 high SDECG expression and immune cell upregulation. Significant differences in SDECGs and DEGs scores were observed between C1 and C2. *In vitro*, ERBB2 expression was significantly elevated in DR cell model. Beta-sitosterol inhibited ERBB2 protein and mRNA expression and reduced IL-1β, VEGF, and ANGPTL6 secretion. ERBB2 inhibition also reduced these biomarkers.

**Conclusion:**

MDBD treats DR by targeting SDECGs, modulating immune responses, and reducing inflammation. Beta-sitosterol and ERBB2 inhibition showed significant therapeutic effects, offering valuable insights for clinical application and future research directions.

## 1 Introduction

Diabetic retinopathy (DR), a major complication of diabetes mellitus (DM), is the leading cause of visual impairment and preventable blindness among working-age adults worldwide (1). It is classified into non-proliferative DR (NPDR) and proliferative DR (PDR), both of which result from hyperglycemia-induced damage to retinal blood vessels, causing swelling, leakage, and eventually bilateral vision loss (2). The global prevalence of DR is estimated to range from 30% to 40%, with developing countries facing higher rates due to limited access to healthcare and resources (3–5). Currently, approximately 93 million people are affected by DR globally (6), and modern lifestyle factors, such as physical inactivity and sedentary behavior, further contribute to its prevalence (7). Both type 1 and type 2 diabetes mellitus (T1DM and T2DM) accelerate the progression of DR, with poor glycemic control, hypertension, hypercholesterolemia, and insufficient physical activity identified as key risk factors (8, 9). Alarmingly, the global incidence of DR among adults is expected to rise to 129.84 million by 2030 and 160.50 million by 2045 (10). Management strategies for DR aim to mitigate pathological microvascular changes and preserve visual acuity (11). Intravitreal agents, such as anti-VEGF therapies like aflibercept and ranibizumab, and corticosteroid implants including dexamethasone and fluocinolone, have shown significant efficacy in reducing vision-threatening complications, particularly diabetic macular edema (DME) and PDR (12). However, anti-VEGF therapies are associated with adverse effects, including macular ischemia, retinal pigment epithelial tears, elevated intraocular pressure, endophthalmitis, retinal vasculitis, and retinal artery occlusion (13). Corticosteroid treatments also pose risks, such as inflammation, cataract formation, vision deterioration, uveitis, ocular discomfort, blurred vision, and retinal detachment, which may lead to lens opacity and vision loss (14). These challenges show the importance of developing new management approaches to reduce complications and improve patient outcomes.

In China, Traditional Chinese Medicine (TCM) has been widely applied in the treatment of diabetic retinopathy (DR), with its efficacy supported by clinical trials. A network meta-analysis has demonstrated that integrating TCM with conventional Western therapies produces better outcomes compared to Western medicine alone (15). The Modified Danggui Buxue Decoction (MDBD), comprising *Astragalus membranaceus* (Fisch.) Bge., *Angelica sinensis* (Oliv.) Diels, and *Panax notoginseng* (Burk.) F.H.Chen. in a 1:5:1 ratio, is recognized for its ability to nourish qi, replenish blood, and resolve stasis, thereby improving DR (16). *Astragalus membranaceus* (Fisch.) Bge., a perennial herb from the Leguminosae family, is a prominent traditional Chinese medicine known for its function in replenishing Qi and elevating Yang in TCM theory (17). The dried root of this herb contains bioactive compounds, such as polysaccharides, flavonoids, and saponins, which exhibit immunomodulatory, anti-inflammatory, and anti-tumor activities (18–20). *Angelica sinensis* (Oliv.) Diels, the dried root of a perennial herb in the Umbelliferae family, has long been used in Chinese medicine for its role in nourishing and invigorating the blood (21). Its therapeutic effects are attributed to mechanisms such as antioxidant activity, regulation of apoptosis, and modulation of inflammatory responses (22–24). *Panax notoginseng* (Burk.) F.H.Chen., commonly known as a traditional chinese medicinal herb, a widely utilized traditional Chinese medicinal herb, has been employed for centuries to promote blood circulation and stop bleeding, making it a vital component in haemostatic and tonic formulations (25). Its pharmacological properties include significant effects on the cardiovascular and immune systems, as well as haemostatic, anti-inflammatory, and anti-tumor activities (26–28). MDBD integrates the synergistic effects of three potent herbs, offering a comprehensive therapeutic approach to addressing blood-related disorders and promoting overall health.

Sudies have demonstrated the compounds extracted from *Astragalus membranaceus* (Fisch.) Bge., *Angelica sinensis* (Oliv.) Diels, and *Panax notoginseng* (Burk.) F.H.Chen. in MDBD may contribute to its benefits for DR. For example, astragaloside I, extracted from *Astragalus membranaceus*, has been shown to reduce renal fibrosis in diabetic kidney disease by inhibiting HDAC3 and TGF-β1, thereby regulating the Klotho/TGF-β1/Smad2/3 pathway (29). Similarly, Angelica polysaccharides from *Angelica sinensis* have demonstrated the ability to alleviate glycemic disorders in T2D KKAy mice by improving gut microbiota composition and function (30). In addition, ginsenoside Rb1, the main active compound from *Panax notoginseng*, has been reported to reduce high glucose-induced podocyte apoptosis and mitochondrial damage by targeting aldose reductase, slowing the progression of diabetic kidney disease (31).

Our previous clinical studies have shown that MDBD effectively improves retinal health in patients with non-proliferative diabetic retinopathy (NPDR). Fundus images revealed reductions in microaneurysms, hemorrhages, and exudations, alongside improvements in visual acuity, both superior to the untreated NPDR or calcium dobesilate group (32–34). Key indicators of blood routine, including White Blood Cell Count (WBC) and Neutrophil Count (NE), liver function indicators Alanine Aminotransferase (ALT) and Aspartate Aminotransferase (AST), and kidney function indicators Blood Urea Nitrogen (BUN), Creatinine (CRE), and Uric Acid (UA), remained within normal ranges, with no significant differences observed after treatment (32, 33). MDBD demonstrates comparable efficacy and a favorable safety profile, showing promise as a reliable and safe treatment option. Furthermore, our previous study revealed that MDBD protects retinal Müller cells from hypoxia-induced apoptosis by inhibiting the ATF4/CHOP pathway, reinforcing its protective role in DR (35). Despite these encouraging results, the mechanisms behind MDBD’s therapeutic effects in DR remain unclear.

**Figure 1:**
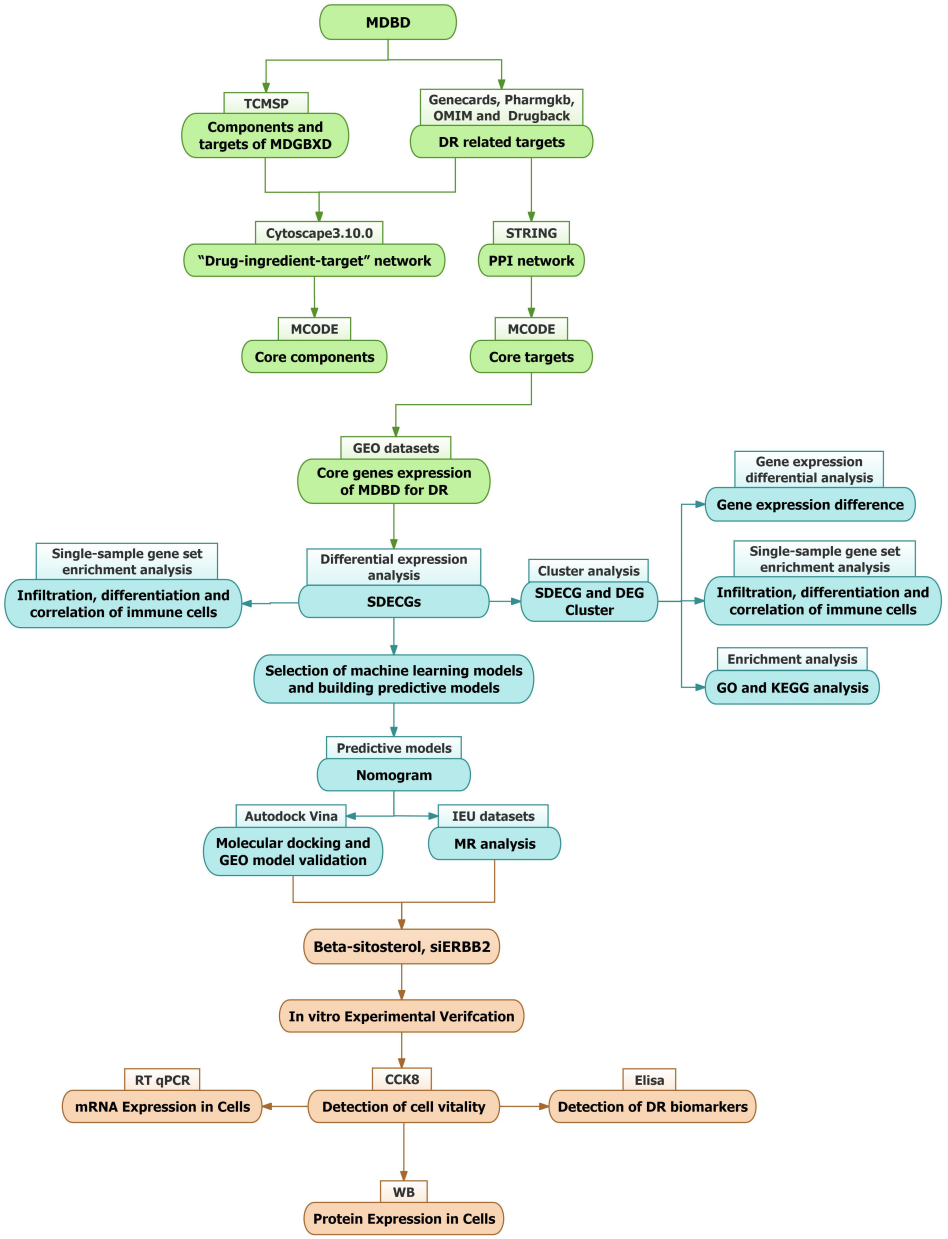
Study flowchart.

This study aims to comprehensively analyze MDBD’s role in DR treatment, employing network pharmacology to identify its key components and therapeutic targets. Differentially expressed core genes (SDECGs) were identified using samples from the GEO database. Validation was conducted through molecular docking, additional GEO datasets, and Mendelian randomization (MR) to elucidate the specific mechanisms and pathways involved. These findings provide a foundation for further experimental validation (**Figure 1**).

## 2 Method

### 2.1 Collection of the components and targets of MDBD

Active constituents and targets of Astragalus membranaceus (Fisch.), Angelica sinensis (Oliv.) Diels, and Panax notoginseng (Burk.) F.H. Chen were identified via the Traditional Chinese Medicine Systems Pharmacology (TCMSP) platform (https://tcmsp-e.com/tcmsp.php). Criteria for filtering included oral bioavailability (OB) of at least 30% and drug-likeness (DL) of at least 0.18. Target validation was subsequently performed utilizing the UniProt database.

### 2.2 Identification of targets associated with DR

Target genes associated with diabetic retinopathy were retrieved by searching Genecards (https://www.genecards.org/), OMIM (https://omim.org/), PharmGKB (https://www.pharmgkb.org/), and Drugbank (https://go.drugbank.com/). The collected target genes were consolidated, and duplicates were eliminated.

### 2.3 Development of the drug-component-target network

The Venn package was employed to identify intersections between active ingredients in MDBD and DR-related targets. Cytoscape V3.10.0 was then used to construct a network, incorporating diseases, drugs, components, and DR-related targets as nodes, with their interrelationships represented as edges. Topological analysis was conducted to identify core components within the network.

### 2.4 Construction of protein-protein interaction network

The intersecting targets’ PPI network was retrieved from the String database (https://string-db.org), specifying Homo sapiens as the species and setting the minimum interaction score at 0.40. Using Cytoscape V3.10.0, a network was constructed with nodes representing diseases, drugs, components, and DR-related targets, while edges depicted their relationships. To pinpoint key components and core PPI targets, the MCODE plugin was applied for clustering and topological analysis.

### 2.5 Acquisition and processing of GEO samples

The keyword ‘Diabetic retinopathy’ guided sample searches in the GEO database (https://www.ncbi.nlm.nih.gov/geo/), with constraints on data type (expression profiles) and species (Homo sapiens). Gene expression and clinical data were extracted to evaluate core gene expression levels related to MDBD treatment for diabetic retinopathy across normal and DR groups.

### 2.6 Differential expression and correlation analysis of core genes

Core gene expression levels were extracted from control and DR groups, followed by differential expression analysis using R packages such as ‘limma’, ‘pheatmap’, and ‘ggpubr’ in R V4.3.3. Results were visualized via box plots and heatmaps, identifying genes with *P* < 0.05 as SDECGs. Perl scripts determined the chromosomal positions of these genes, which were then displayed in circular plots using the R package ‘Rcircos’. The ‘cor’ function calculated and visualized correlation coefficients for each SDECG.

### 2.7 Immune cell infiltration and correlation in DR samples

The CIBERSORT command in R was employed to conduct 1000 simulations, determining the relative abundance of immune cells. A bar graph was used to illustrate immune cell content per sample. Single-sample gene set enrichment analysis (ssGSEA) was performed using R packages ‘GSVA’ and ‘GSABase’ to compare immune cell content differences between normal and DR groups, with results shown in box plots. Correlation testing was conducted between SDECGs and ssGSEA scores, and coefficients were visualized. CIBERSORT results were finalized.

### 2.8 Development of predictive models for DR

Four predictive models, Random Forest (RF), Support Vector Machine (SVM), Generalized Linear Model (GLM), and Extreme Gradient Boosting (XGB), were developed based on SDECG expression data. Feature genes were selected using residual cumulative distribution plots, residual boxplots, and Receiver Operating Characteristic (ROC) curves. The optimal model was constructed incorporating feature genes and their expression levels across normal and DR groups. Decision and calibration curves were formulated to evaluate model accuracy.

### 2.9 MR analysis between feature genes and DR

A two-sample Mendelian Randomization (MR) analysis was conducted to explore the causal link between feature genes and DR risk, with SNPs designated as instrumental variables (IVs). Feature gene SNPs were sourced from the Integrative Epidemiology Unit (IEU) database (https://gwas.mrcieu.ac.uk/) as exposure variables, while DR-related SNPs were outcome variables. The “TwoSampleMR” package facilitated MR analysis, employing the inverse variance weighted (IVW) method to assess the correlation between feature gene expression levels and DR risk. Heterogeneity was examined using Cochran’s Q statistic, with *P* < 0.05 indicating heterogeneity in IVW results. MR-Egger regression and MR-PRESSO analysis were used to evaluate potential horizontal pleiotropy, with *P* < 0.05 suggesting its presence.

### 2.10 Molecular docking of core compounds with feature genes

The 3D structures of the core components and feature genes of MDBD were retrieved from the PubChem (https://pubchem.ncbi.nlm.nih.gov) and Protein Data Bank (PDB) (http://www.rcsb.org/) databases. Molecular docking was performed using Autodock Vina to preliminarily verify the interaction between the core network pharmacological components and feature genes, and the top four docking combinations were selected and visualized with Pymol. Differential expression analysis of GEO datasets was performed to identify key genes between the normal and DR groups, providing additional validation for the molecular mechanisms.

### 2.11 Cluster analysis of SDECGs

The R package ‘ConsensusClusterPlus’ was utilized to categorize DR samples based on SDECG expression, employing the k-means clustering technique with Euclidean distance, allowing for up to nine clusters. Comparative analysis of clustering outcomes was performed using heatmaps and boxplots to evaluate expression levels. Principal Component Analysis (PCA) was conducted to discern inter-cluster differences. Subsequent ssGSEA analysis on SDECG clusters generated bar plots to depict immune cell content variations across clusters. Gene Ontology (GO) and Kyoto Encyclopedia of Genes and Genomes (KEGG) enrichment analyses were executed using GMT files from the GSEA platform (http://www.gsea-msigdb.org/). Gene Set Variation Analysis (GSVA) was conducted in R V4.3.3 to assess enriched gene expression across clusters. Differential gene expression analysis, adhering to|logFC|>1 and adj.P-Value<0.05, was followed by Venn diagram visualization to identify differentially expressed genes (DEGs) among clusters.

### 2.12 Enrichment analysis of DEGs among SDECG clusters

The DEGs among SDECG clusters underwent GO enrichment analysis covering biological processes (BP), molecular functions (MF), and cellular components (CC), alongside KEGG pathway enrichment. These analyses were executed using R packages such as “clusterProfiler” and “enrichplot” in R V4.3.3, with a screening threshold of P-value <0.05. Results were depicted through circular plots and bar graphs.

### 2.13 Cluster analysis of DEGs

An additional cluster analysis, based on DEG expression, was performed using the methodology described in Section 2.11, selecting the DEG cluster with the highest precision. The DEG clustering results were used to compare DEG expression levels across clusters, SDECG expression variations, and immune cell content differences among clusters. These findings were visualized using heatmaps and box plots.

### 2.14 Differential analysis of SDECGs and construction of alluvial diagrams

Scores for SDECGs were computed for each sample based on expression levels, utilizing PCA by incorporating PC1 and PC2. Differential analysis of SDECGs and DEGs clustering scores was executed using R packages such as “limma” and “ggpubr” in R V4.3.3. Box plots were generated to depict SDECGs scores across clustered samples. Additionally, the “ggalluvial” package facilitated the creation of alluvial diagrams to illustrate relationships and processes among SDECGs clusters, DEGs clusters, and samples with varying SDECGs scores.

### 2.15 Preparation for beta-sitosterol

Beta-sitosterol (HY-N0171A, MedChemExpress, USA) was dissolved in DMSO (D8371, Solarbio, USA). The solution was subsequently diluted in complete culture medium for mouse retinal Müller cells (CM-M117, Procell, China) to prepare various concentrations as required for the experiments. Fresh solutions were prepared immediately prior to use.

### 2.16 Cell culture and DR cell model induction

The mouse retinal Müller cells (MMCs) (CP-M117, Procell, China) were purchased from Pricella Biotechnology Co.,Ltd. The cells were cultured in in complete culture medium for mouse retinal Müller cells (CM-M117, Procell, China), and maintained at 37.0 °C with a CO_2_ concentration of 5% in a cell culture incubator. When the confluence of MMCs reached over 80%, they were passaged using trypsin (2.5% EDTA) (SH30042.01, HyClone, USA).

We induced DR cell model according to our previous study (35), utilizing a hypoxia-induced injury approach to simulate the conditions encountered in DR. MMCs were cultured under hypoxic conditions by placing them in a hypoxia chamber with a controlled oxygen concentration of 1% for a duration of 48 hours. Beta-sitosterol were used to incubated MMCs for 24h. The cells were harvested for subsequent analysis.

### 2.17 siERBB2 transfection

The RiboFECT™ CP transfection kit (C10511-05, RiboPharm, China) facilitated cell transfection in a 6-well plate. A solution of siERBB2 was prepared by mixing 3μl of 30μM stock with 120μl of 1X riboFECT™ CP Buffer, followed by the addition of 12μl of riboFECT™ CP reagent to form the transfection complex. This complex was combined with an antibody-free complete culture medium and gently agitated. Subsequent treatments led to incubation of the plates at 37 °C in a CO_2_ incubator for 24 hours prior to assays.

### 2.18 Evaluation of cell viability using CCK-8 assay

Cell viability assessment was conducted using the Cell Counting Kit-8 (CCK-8) (K009-1000T, Zeta-life, USA). MMCs were exposed to various concentrations of Beta-sitosterol in medium at 37 °C for 24 hours following hypoxia induction. Post medium removal, 10 μL of CCK-8 solution was introduced to each well and incubated in a CO_2_ incubator for 1 hour. Absorbance was recorded at 450 nm utilizing a microplate reader.

### 2.19 Real-time quantitative PCR

Total RNA extraction from MMCs was performed using TRIzol (DP424, Tiangen, China). cDNA synthesis followed with the RevertAid First Strand cDNA Synthesis Kit (K1622, Thermo, USA). RT-qPCR was employed to evaluate mRNA expression using 2x SYBR Green qPCR Master Mix (B21203, Selleck, USA) on a CFX96™ real-time system (Bio-Rad, USA). Relative expression levels of target genes were determined via the 2^-ΔΔCt method, based on obtained Ct values. Primers for analyzed genes included:

Erb-B2 Receptor Tyrosine Kinase 2 (ERBB2): forward, 5’-CTGTGTGACCACCTGCCCCTAC-3’, and reverse, 5’-TGCCCAGACCATAGCATACTCC-3’; Beta-actin (β-actin): forward, 5’-AGGTCATCACTATTGGCAACGAG-3’, and reverse, 5’-TTGGCATAGAGGTCTTTACGGAT-3’.

### 2.20 Western blotting

MMC lysis was achieved using the RIPA buffer (R0020, Solarbio, USA) to facilitate protein extraction for subsequent analyses. Quantification of proteins in the lysis supernatant was conducted employing the BCA Protein Assay Kit (P0011, Beyotime, China). Proteins were separated via SDS polyacrylamide gel electrophoresis (SDS-PAGE) utilizing the SDS-PAGE Gel Kit (P1200, Solarbio, USA), followed by transfer onto polyvinylidene fluoride (PVDF) membranes (IPVH00010, Millipore, Germany). After blocking and washing, the membranes underwent incubation with a primary antibody for 20 hours, succeeded by a 2-hour incubation with a secondary antibody. The proteins on PVDF membranes were processed with BeyoECL Plus (P0018S, Beyotime, China) to enhance chemiluminescence. Antibodies employed for Western blotting included ERBB2 (18299-1-AP, Proteintech), and glyceraldehyde-3-phosphate dehydrogenase (GAPDH) (UM4002, UtiBody, China).

### 2.21 Enzyme-linked immunosorbent assay

The quantification of interleukin-1β (IL-1β), vascular endothelial growth factor (VEGF), and angiopoietin-like 6 (ANGPTL6) in Müller cells (MMCs) was performed using specific enzyme-linked immunosorbent assay (ELISA) kits (MM-0040M1, MM-0128M1, MM-48029M1, Meimian, China). ELISA assays were conducted strictly following the manufacturer’s protocols to ensure accuracy and reproducibility. Absorbance measurements were taken at 450 nm using a calibrated microplate reader, and the concentrations were calculated by comparing the absorbance values to standard curves generated during the assay.

### 2.22 Statistical analysis

The statistical analysis was conducted using GraphPad Prism 10 software (GraphPad Software, USA). Quantitative data were expressed as mean ± standard error of the mean (SEM). For comparisons among multiple groups, one-way analysis of variance (ANOVA) was applied, followed by Fisher’s LSD test or Tukey’s *post hoc* test for pairwise comparisons. Differences between two groups were analyzed using the Student’s t-test. Statistical significance was determined by a Type I error probability (α) of less than 5%, with significance levels presented as *P* < 0.05, *P* < 0.01, or *P* < 0.001 to denote varying degrees of statistical difference.

## 3 Results

### 3.1 Constituents and targets of MDBD

Active constituents and targets of Angelica sinensis, Astragalus membranaceus, and Panax notoginseng were acquired via the TCMSP database. Following the elimination of duplicates and irrelevant entries, 30 distinct active components and 1207 associated targets were identified (**Supplementary Table S1**).

### 3.2 Targets associated with DR

**Figure 2:**
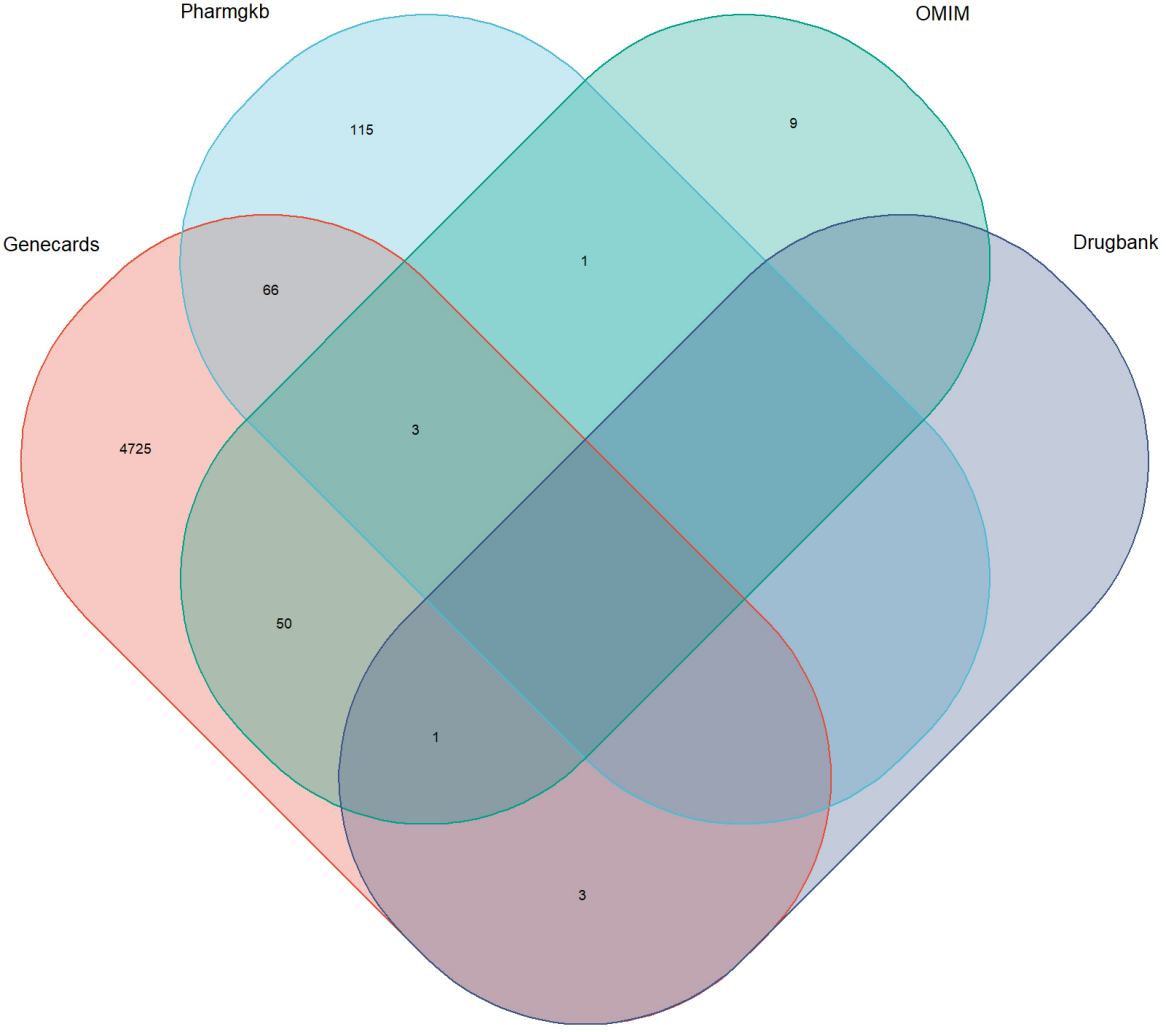
Targets associated with diabetic retinopathy in multiple databases. The overlapping regions represent duplicates.

Targets numbering 4848, 185, 64, and 4 were sourced from Genecards, Pharmgkb, OMIM, and Drugbank, respectively. After duplicate removal, a total of 4973 DR-related targets were obtained (**Figure 2**).

### 3.3 Network of “drug-component-target”

**Figure 3:**
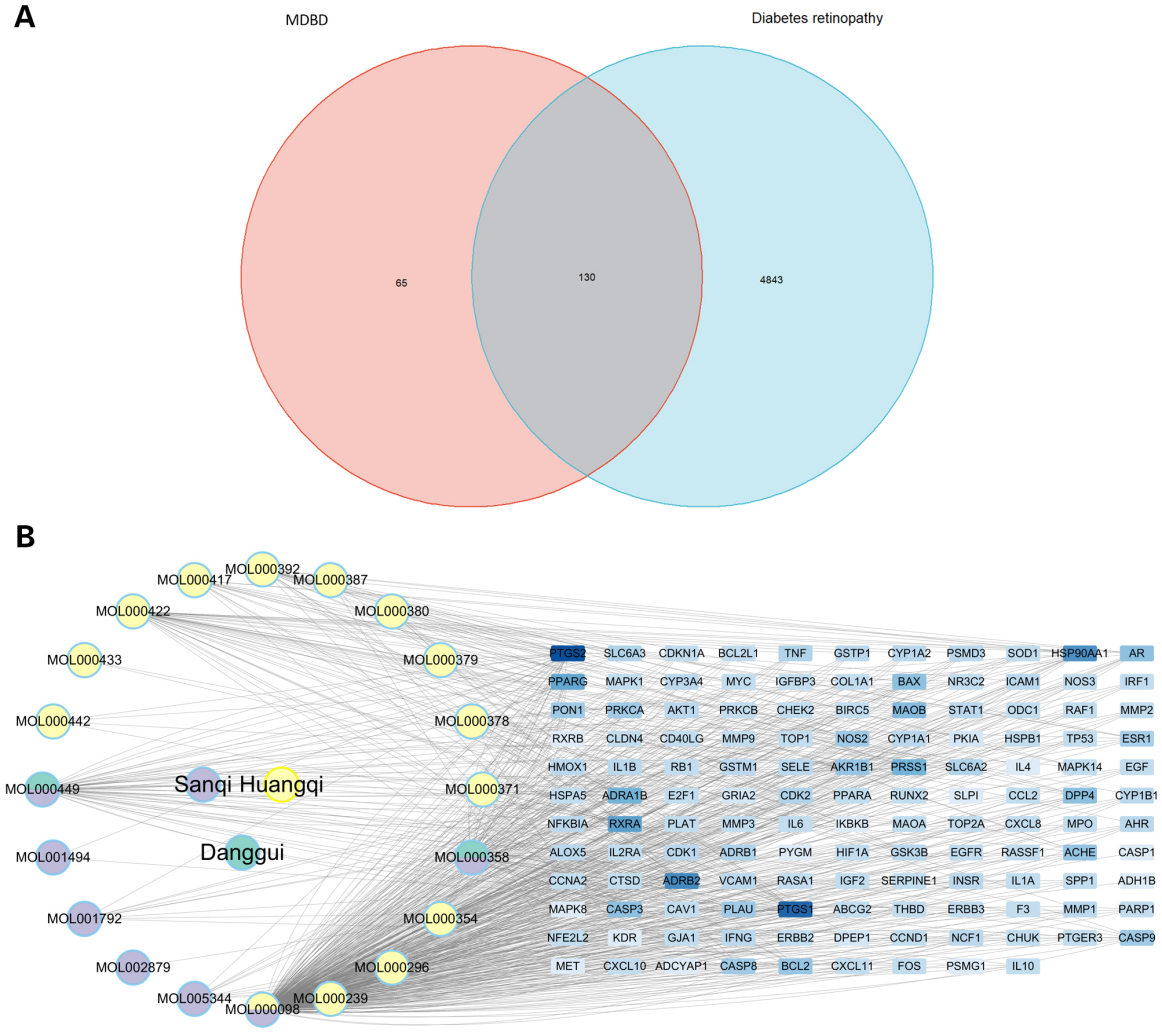
Drug-component-target network. **(A)** Venn diagram of intersection between compounds in MBDB and DR-related targets. **(B)** Interaction network of drug-component-target. This network comprises three traditional Chinese medicines, encompassing 20 compounds and 130 targets. MBDB, Modified Danggui Buxue Decoction.

The intersection between each active ingredient in MDBD and DR-related targets was determined using the venn package, resulting in 130 targets directly linked to drug and disease (**Figure 3A**). Topological analysis was conducted to pinpoint core components within the network (**Figure 3B**).

### 3.4 PPI network

**Figure 4:**
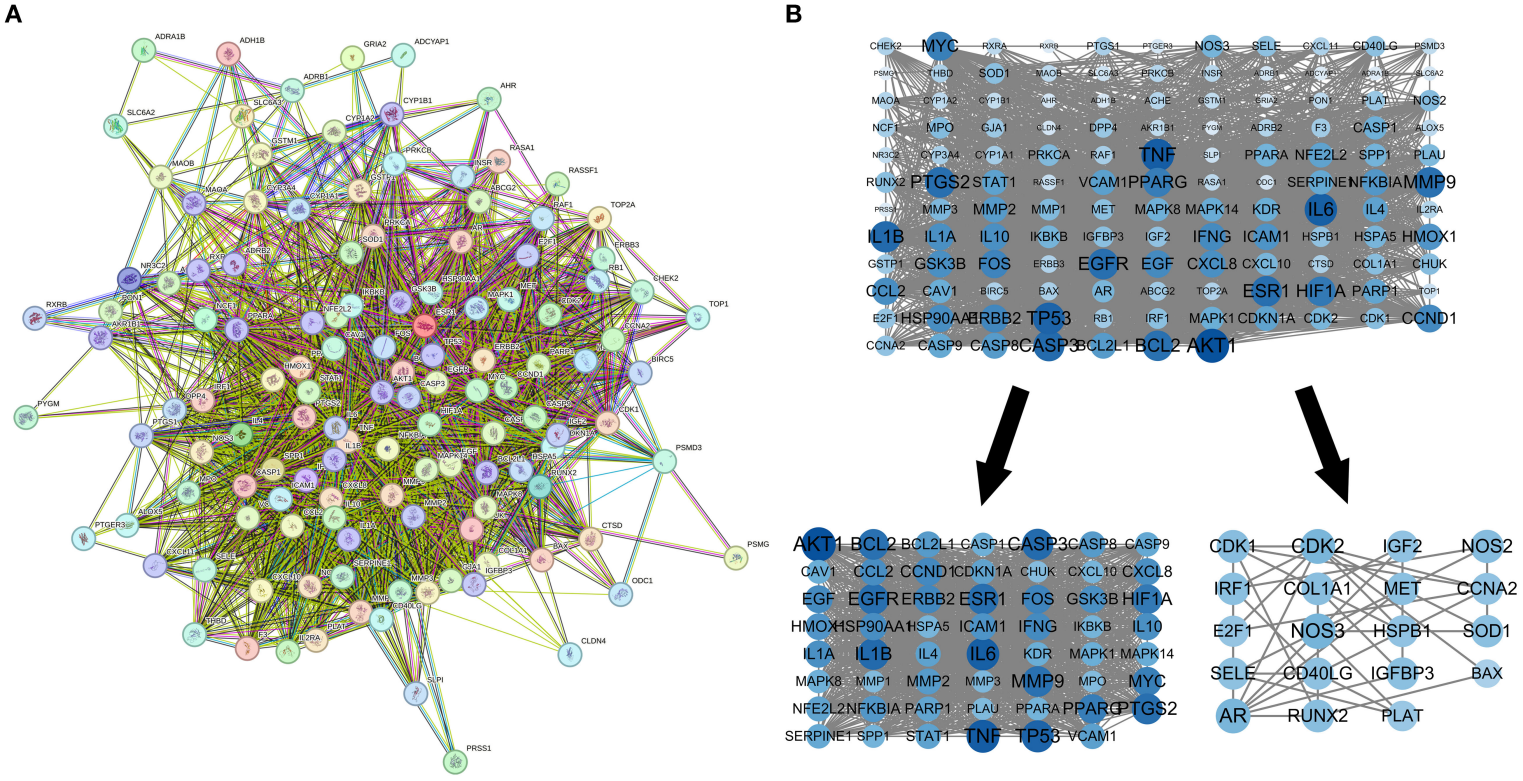
PPI network for modified danggui buxue decoction. **(A)** Protein-protein interaction network for diabetic retinopathy targets. **(B)** Sub-networks highlighting key protein clusters. The protein-protein interaction network, based on targets for diabetic retinopathy, is illustrated. Nodes symbolize distinct proteins, while edges indicate protein associations, with line thickness reflecting data support strength.

The PPI network of intersecting targets was extracted from the String database, with Homo sapiens specified as the species. The network comprised 80 nodes and 1318 edges, with an average node degree of 35.5. The MCODE plugin facilitated clustering of the PPI network to identify core PPI targets, yielding two clustered networks with a minimum interaction score of 0.40. Seventy-four core targets were identified, including MAPK8, CASP9, IL1A, IFN, EGFR, IL1B, CDKN1A, SOD1, ERBB2, NOS3, and CD40LG, which are deemed significant for MDBD treatment of DR (**Supplementary Table S2**; **Figure 4**).

### 3.5 Sample acquisition in GEO datasets

The keyword “diabetic retinopathy” was utilized to extract samples from the GEO database, with restrictions on data type and biological species. Gene expression and clinical data were obtained to assess the expression levels of core genes in MDBD-treated DR within normal and DR cohorts. Two datasets were selected: GSE160306, GPL20301. Macular tissue analysis was performed across various clinical stages: DR for diabetic retinopathy; NPDR for non-proliferative diabetic retinopathy; PDR for proliferative diabetic retinopathy; DME for diabetic macular edema, where elevated scores denote more severe lesions. The study included 20 normal samples and 39 DR samples.

### 3.6 Variations in core gene expression, chromosomal localization, and expression correlation of SDECGs

**Figure 5:**
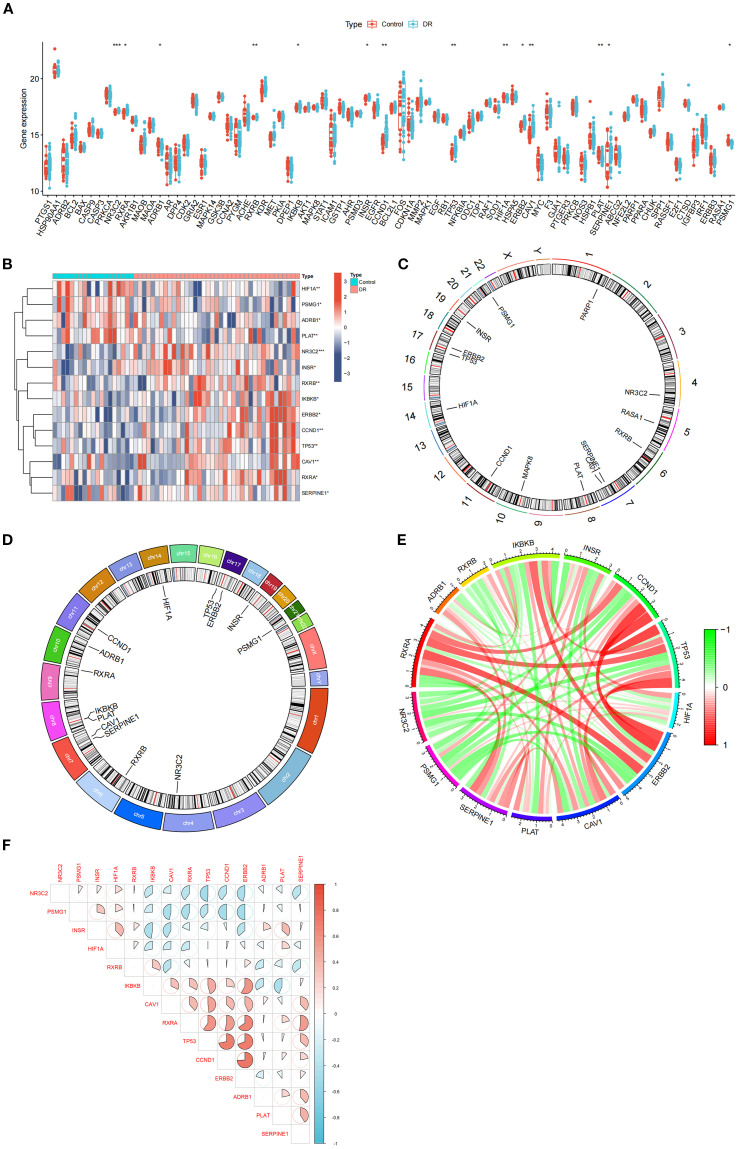
Gene Expression Analysis. **(A)** Box plot of core gene expression. The horizontal axis is core genes, and the vertical axis is gene expression level. Data presented as mean ± SEM. *P<0.05; **P<0.01; ***P<0.001. **(B)** Heat map of SDECGs expression levels. Red presents upregulated genes, and blue presents downregulated genes. Data presented as mean ± SEM. *P<0.05; **P<0.01; ***P<0.001. **(C)** Circular plot of chromosomal locations of SDECGs; **(D)** Circos plot of 14 SDECGs. **(E)** Circular plot of the correlation among SDECGs, with red indicating positive and green negative correlations. **(F)** Correlation matrix between SDECGs. Red presents positive correlation, and blue presents negative correlations.

Seventy-four core genes of MDBD were identified through network pharmacology analysis (**Supplementary Table S2**). Expression levels were extracted from both the control and DR groups, followed by differential expression analysis. Fourteen genes, including NR3C2, PSMG1, INSR, HIF1A, RXRB, IKBKB, CAV1, RXRA, TP53, CCND1, ERBB2, ADRB1, PLAT, and SERPINE1, were validated as SDECGs core genes in human samples. Except for HIF1A, PSMG1, ADRB1, and PLAT, which exhibited high expression in the control group, the remaining genes were highly expressed in the DR group (**Figures 5A, B**). Chromosomal positions of MDBD core genes were mapped in RCircos (**Figures 5C, D**). Correlation analysis among SDECGs in DR samples revealed strong correlations, encompassing both positive and negative relationships (**Figures 5E, F**).

### 3.7 Immune cell infiltration, differentiation, and correlation in control and DR samples

**Figure 6:**
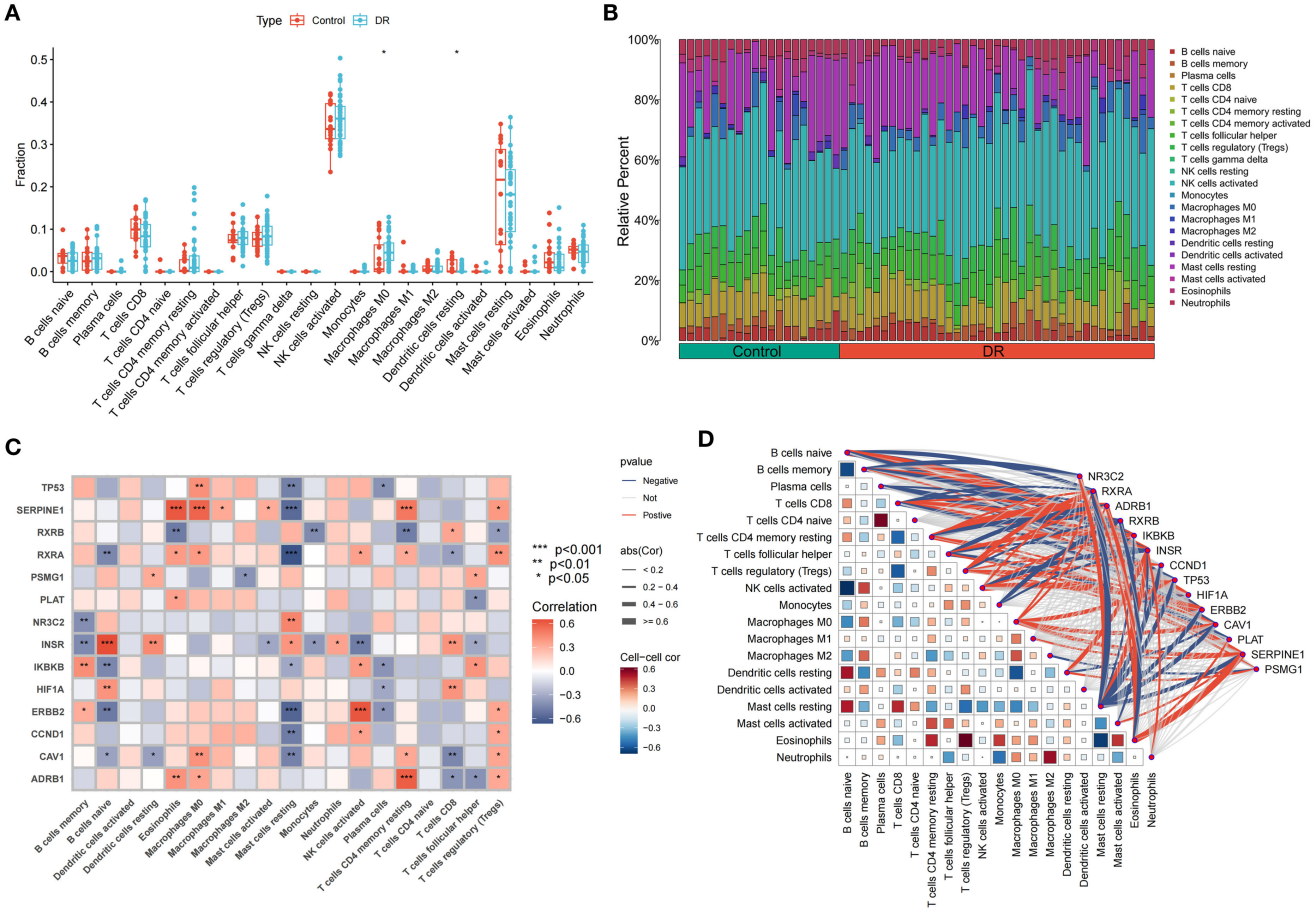
Immune Cell Infiltration Analysis. **(A)** Bar plot of differences in immune cell fraction between control and DR group. The horizontal axis is immune cells, and the vertical axis is immune cell fraction. The data are presented as the mean ± SEM. *P<0.05. **(B)** Distribution proportion of immune cells within samples. **(C)** Heatmap of correlations between immune cell infiltration and SDECG. The horizontal axis is SDECGs, and the vertical axis is immune cells. Red presents positive correlation, and blue presents negative correlations. The data are presented as the mean ± SEM. *P<0.05; **P<0.01; ***P<0.001. **(D)** Network and heatmap of correlations between immune cell infiltration and SDECG. Darker colors indicate stronger correlations between immune cells; thicker lines indicate stronger gene–immune cell correlations. Red presents positive correlation, and blue presents negative correlations.

Single-sample gene set enrichment analysis (ssGSEA) was conducted to compare immune cell levels between control and DR groups (**Figure 6A**), identifying statistically significant immune cells such as macrophages M0 with elevated expression in the DR group and resting dendritic cells with high expression in the control group. Immune cell infiltration analysis determined the types and levels of immune cells in each sample, visualizing these levels (**Figure 6B**). The intersection of SDECGs with ssGSEA scores for immune cell correlation testing indicated that correlations between SDECGs and immune cells included both positive and negative relationships (**Figures 6C, D**). Among the immune cells significantly associated with SDECGs (*P* < 0.05), dendritic cells resting, eosinophils, macrophages M0, macrophages M1, neutrophils, activated NK cells, resting CD4 memory T cells, CD8 T cells, and regulatory T cells (Tregs) were predominantly positively correlated with the relevant SDECGs. Conversely, memory B cells, naive B cells, macrophages M2, resting mast cells, monocytes, plasma cells, and follicular helper T cells were mainly negatively correlated with the relevant SDECGs.

### 3.8 Selection of machine learning models and construction of predictive models for DR treatment

**Figure 7:**
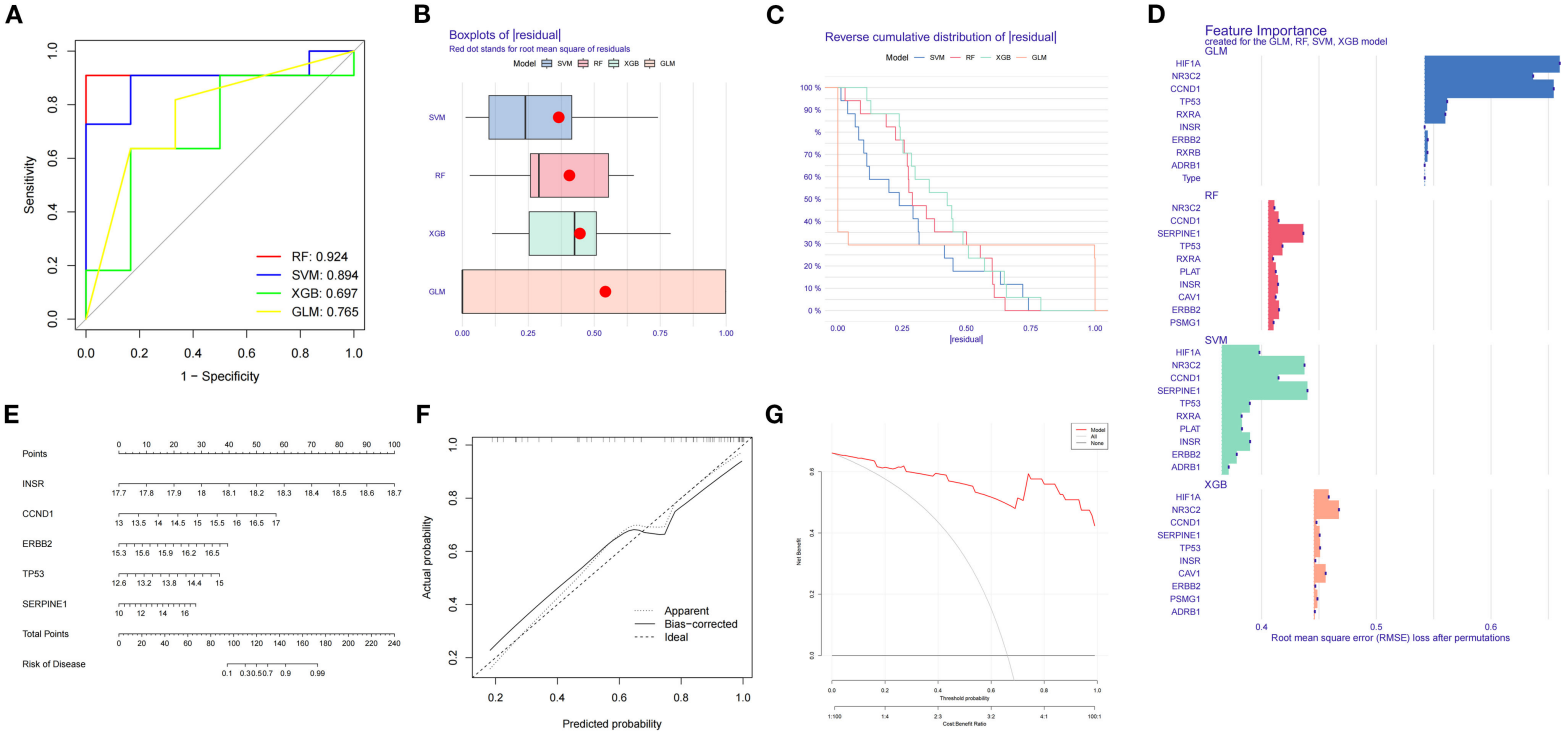
**(A)** ROC curves and AUC for RF, SVM, GLM, XGB. **(B)** Box plots of residuals distribution. The horizontal axis is residuals, and the vertical axis is models. Red dot denotes the root mean square of residuals. **(C)** Reverse cumulative residual distribution. The horizontal axis is residuals, and the vertical axis is reverse cumulative proportion (%). **(D)** Feature importance for RF, SVM, GLM, XGB. The horizontal axis is root mean square error loss after permutations, and the vertical axis is feature genes. The larger the value, the more important the feature gene. **(E)** Nomogram predicts disease risk using key features. **(F)** Calibration plot: predicted vs. actual probability. **(G)** Performance metrics include RMSE for model accuracy.

Expression data of SDECGs served to construct four predictive models: RF, SVM, GLM, and XGB. Analysis of residual box plots, ROC curves, and reverse cumulative distribution plots demonstrated that the RF model achieved the highest accuracy, featuring the largest area under the ROC curve and minimal residuals and reverse cumulative values (**Figures 7A–C**). The RF model, selected for further development, facilitated the construction of a predictive model to derive feature importance scores for the genes. Results indicated that SERPINE1 had the highest score, with gene importance ranked as SERPINE1, TP53, ERBB2, CCND1, INSR, PLAT, NR3C2, CAV1, PSMG1, and RXRA (**Figure 7D**). The top five genes, CCND1, ERBB2, INSR, TP53, and SERPINE1, were utilized for nomogram construction (**Figure 7E**). Finally, decision and calibration curves assessed the predictive model’s accuracy, demonstrating high accuracy based on positive outcomes (**Figures 7F, G**).

### 3.9 Mendelian randomization analysis of feature genes and DR

**Figure 8:**
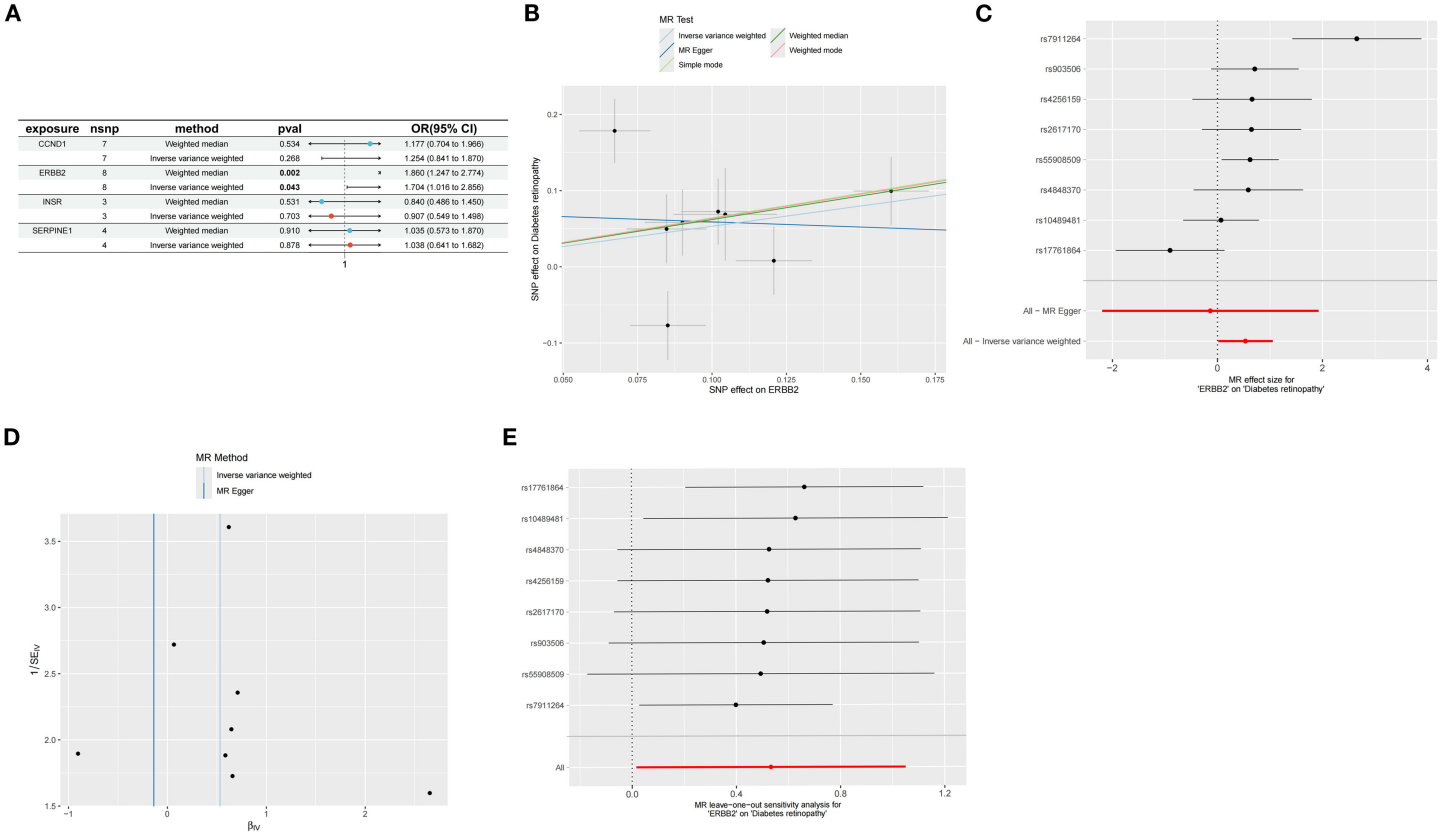
Mendelian randomization analysis of feature genes and DR. **(A)** Mendelian randomization analysis of five top feature genes. **(B)** SNP effects on ERBB2 and diabetic retinopathy. **(C)** Forest plot of MR effect sizes for ERBB2 on diabetic retinopathy. **(D)** Funnel plot for MR analysis of ERBB2 on diabetic retinopathy. **(E)** Leave-one-out sensitivity analysis for MR of ERBB2 on diabetic retinopathy.

MR analysis was executed to investigate the causal relationship between specific feature genes and DR, with SNPs defined as IVs. SNPs from signature genes were utilized as exposure factors, while SNPs related to DR served as outcome factors. Information on the SNPs for the four highlighted genes CCND1, ERBB2, INSR, and SERPINE1 can be found in **Supplementary Table S3**. No SNPs were identified as weak IVs. Due to insufficient data, MR analysis could not be performed on TP53. IVW analysis indicated that ERBB2 is linked to an increased risk of DR (Odds Ratio [OR]=1.70, 95% CI: 1.016 to 2.856, *P* = 0.04), whereas the other three genes did not exhibit a significant causal relationship with DR (**Figure 8A**; **Supplementary Table S4**). The IVW results confirmed ERBB2 as a risk factor for DR, with consistent effect directions observed in the weighted median analysis (OR = 1.860, 95% CI: 1.247-2.774, *P* = 0.002), as demonstrated by both scatter and forest plots (**Figures 8B, C**).

Cochran’s Q test was employed to evaluate study heterogeneity, revealing significant heterogeneity among the selected instrumental variables (*P* = 0.003) (**Figure 8D**; **Supplementary Table S5**). Consequently, IVW with random effects was applied in all MR analyses to mitigate heterogeneity impact. Despite Cochran’s Q test detecting some heterogeneity, MR-Egger analysis showed minimal influence from horizontal pleiotropy (*P*>0.05) (**Figure 8D**; **Supplementary Table S4**), indicating no directional pleiotropy and affirming stable causal relationships. The leave-one-out analysis demonstrated consistent MR analysis results upon sequential SNP removal, indicating robustness and negligible impact on the overall findings (**Figure 8E**).

### 3.10 Molecular docking analysis and validation of GEO datasets

**Figure 9:**
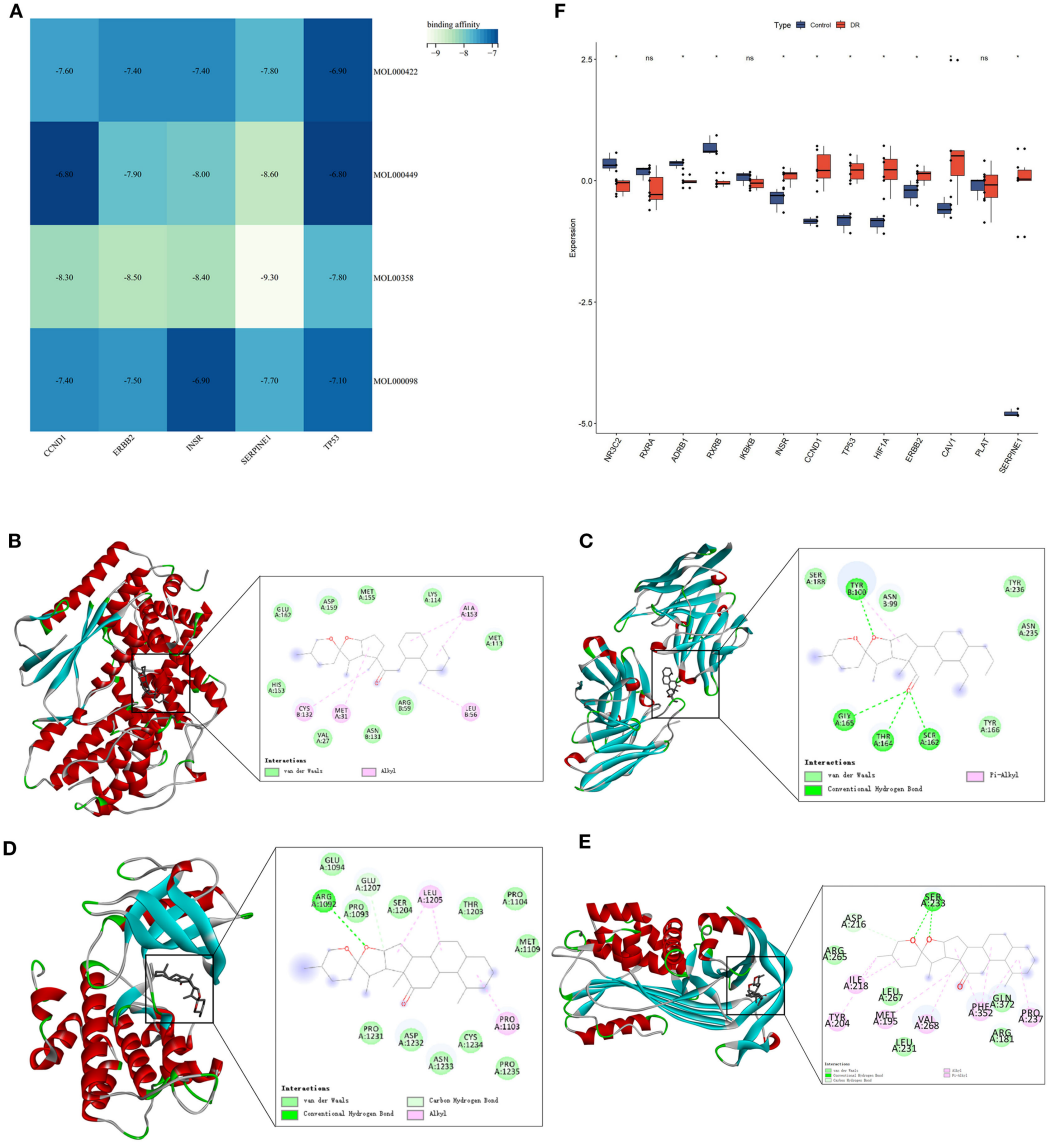
Molecular Docking analysis of feature genes and core components of MDBD and validation of GEO datasets. **(A)** Heatmap of molecular docking analysis of feature genes and core components of MDBD. The horizontal axis is five top feature genes, and the vertical axis is core components. Darker color denotes weaker affinity and lighter color denotes stronger affinity. **(B–E)** Molecular docking of Beta-sitosterol with CCND1, ERBB2, INSR, and SERPINE1. **(F)** Differential expression analysis between the control and DR groups. The horizontal axis is feature genes, and the vertical axis is gene expresion level. The data are presented as the mean ± SEM. *P<0.05.

Autodock Vina was utilized for molecular docking to preliminarily validate interactions between core pharmacological components and feature genes. The optimal docking combinations were selected and visualized using Pymol. Through molecular docking analysis of feature genes and core components of MDBD, it was observed that the binding energy for all docking combinations was below -6.0 kcal/mol, suggesting stable structures could be formed between feature genes and core components (**Figure 9A**; **Supplementary Table S6**).

**Figures 9B–E** showed the top docking combinations of four key gene proteins with the core components of MDBD, while **Figure 9F** presented differential expression analysis of GEO datasets between the control and DR groups. Beta-sitosterol exhibited high binding affinity with CCND1, ERBB2, INSR, and SERPINE1. Alkyl interactions with CCND1 residues CYS132, MET31, LEU56, and ALA153, along with van der Waals forces with CCND1 residues GLU162, ASP159, MET155, LYS114, MET113, HIS153, VAL27, ASN131, and ARG59 were noted, with a docking energy of -8.3 kcal/mol (**Figure 9B**). Conventional hydrogen bonds were formed with ERBB2 residues TYR100, GLY165, THR164, and SER162, along with van der Waals interactions with ERBB2 residues SER188, ASN99, TYR236, ASN235, and TYR166, and a pi-alkyl interaction with TYR100, resulting in a docking energy of -8.5 kcal/mol (**Figure 9C**). Beta-sitosterol also established a conventional hydrogen bond with INSR residues ARG1092 and a carbon hydrogen bond with GLU1207. Additional van der Waals interactions with residues GLU1094, PRO1093, SER1204, THR1203, PRO1104, MET1109, PRO1235, CYS1234, ASN1233, ASP1232, and PRO1231, as well as alkyl interactions with LUE1205 and PRO1103, were observed, with a docking energy of -8.4 kcal/mol (**Figure 9D**). Furthermore, beta-sitosterol demonstrated high binding affinity for SERPINE1 residues SER233 through conventional hydrogen bonding and ASP216 through carbon hydrogen bonding. Van der Waals interactions with SERPINE1 residues ARG265, LEU267, LEU231, ARG181, and GLN273, along with pi-alkyl interactions with residues ILE218, TYR204, MET195, VAL268, PHE352, and PRO237, were also noted, with a docking energy of -9.3 kcal/mol (**Figure 9E**).

### 3.11 Cluster analysis of SDECGs and inter-cluster comparisons

**Figure 10:**
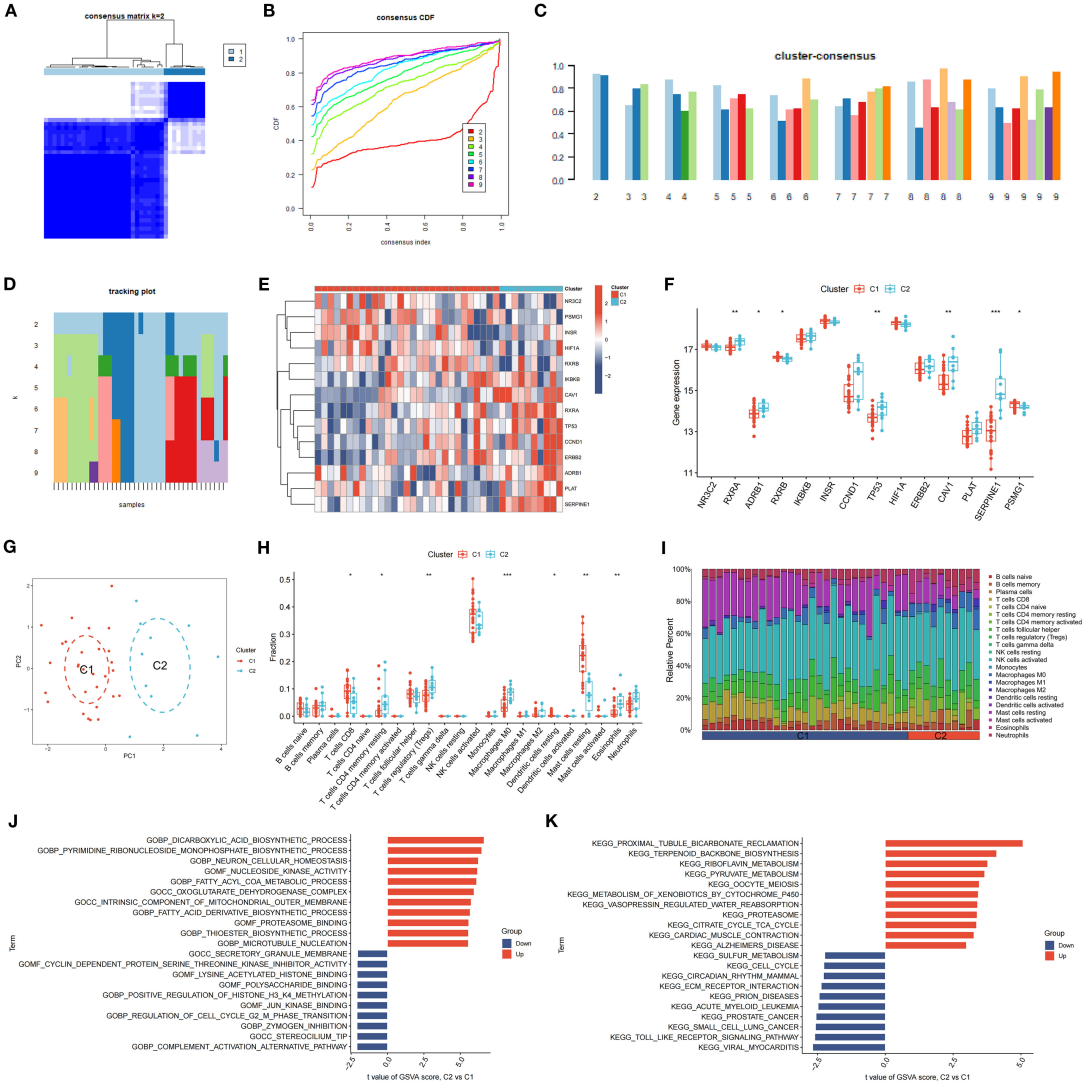
Clustering and Expression Analysis of SDECGs. **(A)** A heatmap of the consensus matrix for the clustering of SDECGs across samples. **(B)** The cumulative distribution functions derived from the consensus matrix of SDECGs clustering. **(C)** The cluster consensus plot of mean consensus scores across different clusters. The horizontal axis shows the number of clusters (k = 2–9), and the vertical axis shows the mean consensus score. Colors represent the respective clusters. **(D)** The tracking plot of mean consensus scores for diverse clusters. **(E)** Heatmap of the expression of SDECGs between C1 and C2. Red presents upregulated genes, and blue presents downregulated genes. **(F)** The box plot of he expression differences of SDECGs between C1 and C2. The horizontal axis is SDECGs, and the vertical axis is gene expresion level. The data are presented as the mean ± SEM. *P<0.05; **P<0.01; ***P<0.001. **(G)** The PCA scatter plots of SDECGs between C1 and C2. **(H, I)** ssGSEA comparison of immune cells between C1 and C2. The horizontal axis is immune cells, and the vertical axis is immune cell fraction in figure 10H. The data are presented as the mean ± SEM. *P<0.05; **P<0.01; ***P<0.001. **(J, K)** GO and KEGG gene set enrichment analysis by GSEA of C1 and C2.

K-means clustering, utilizing Euclidean distance and a maximum of nine clusters, was employed to categorize DR samples based on SDECG expression. The analysis revealed two distinct clusters, achieving optimal accuracy (**Figures 10A, B**) and demonstrating high cluster stability (**Figures 10C, D**). Subsequent examination of SDECG expression in these clusters identified significantly elevated levels of NR3C2, PSMG1, INSR, HIF1A, and RXRB in Cluster C1 (*P* < 0.05, *P* < 0.01, *P* < 0.001), whereas IKBKB, CAV1, RXRA, TP53, CCND1, ERBB2, ADRB1, PLAT, and SERPINE1 showed increased expression in Cluster C2 (*P* < 0.05, *P* < 0.01, *P* < 0.001) (**Figures 10E, F**). Principal component analysis (PCA) results indicated a discernible separation between Clusters C1 and C2, with Cluster C1 exhibiting a higher density (**Figure 10G**). To assess immune cell level variations between C1 and C2, ssGSEA was conducted, identifying statistically significant immune cell populations. Notably, Cluster C1 exhibited higher fractions of CD8+ T cells, resting dendritic cells, and resting mast cells (*P* < 0.05, *P* < 0.01, *P* < 0.001), while Cluster C2 had elevated levels of resting CD4+ memory T cells, regulatory T cells (Tregs), M0 macrophages, and eosinophils (*P* < 0.05, *P* < 0.01, *P* < 0.001) (**Figure 10H**). Immune cell infiltration analysis further characterized the immune cell types and levels in each cluster, visually depicted in **Figure 10I**.

GSVA revealed that terms related to the dicarboxylic acid biosynthetic process, pyrimidine ribonucleoside monophosphate biosynthetic process, neuron cellular homeostasis, nucleoside kinase activity, fatty acyl-CoA metabolic process, and oxoglutarate dehydrogenase complex were upregulated in C2 compared to C1. Conversely, terms associated with the secretory granule membrane, cyclin-dependent protein serine/threonine kinase inhibitor activity, lysine acetylated histone binding, polysaccharide binding, and positive regulation of histone H3 K4 methylation were downregulated (**Figure 9J**). KEGG analysis indicated that pathways such as proximal tubule bicarbonate reclamation, terpenoid backbone biosynthesis, riboflavin metabolism, pyruvate metabolism, oocyte meiosis, and xenobiotic metabolism by cytochrome P450 were upregulated in C2 relative to C1, whereas sulfur metabolism, cell cycle, circadian rhythm, ECM receptor interaction, and prion diseases were downregulated (**Figure 10K**).

### 3.12 DEGs enrichment analysis across SDECG clusters in DR samples

**Figure 11:**
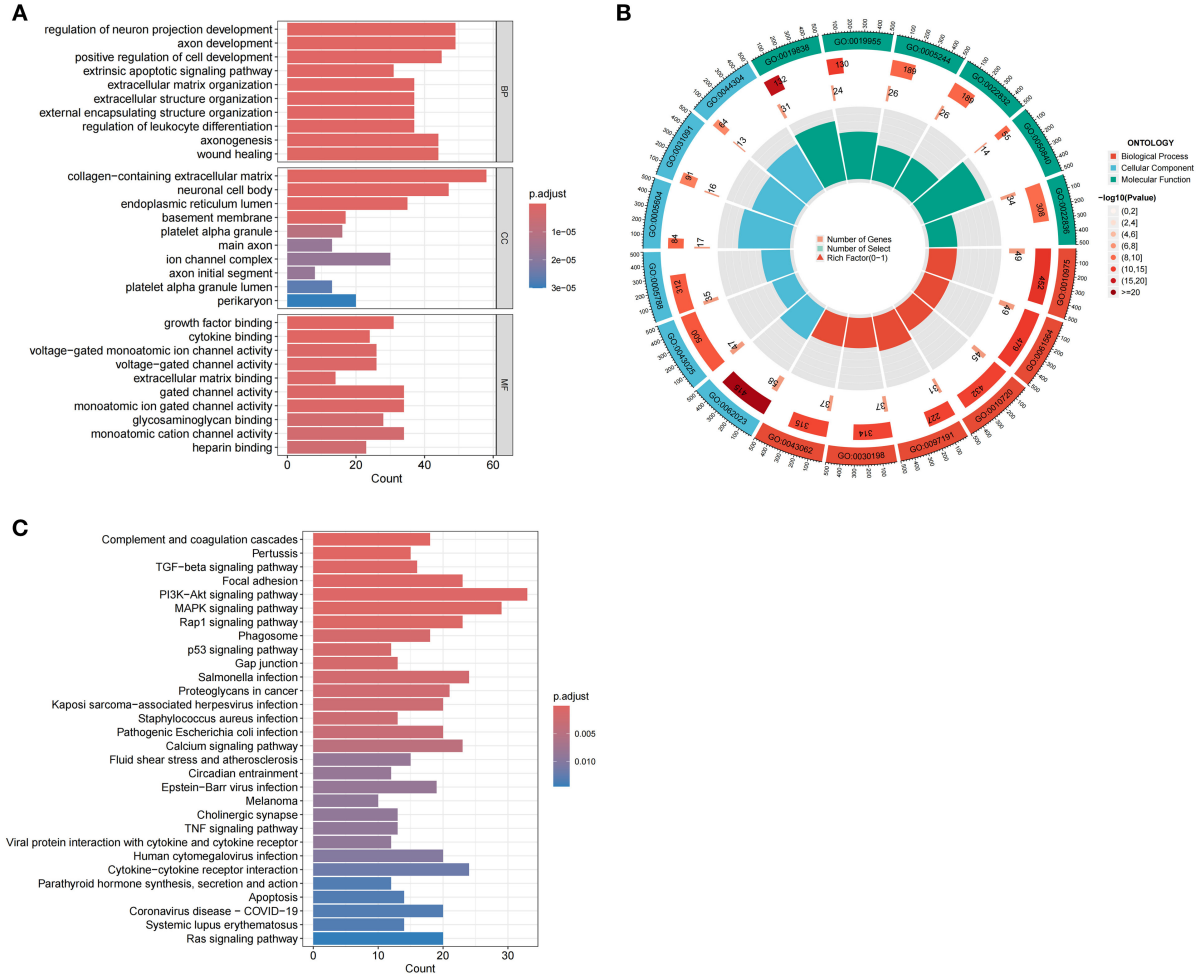
Enrichment Analysis of DEGs Using GO and KEGG. **(A)** GO enrichment analysis of DEGs. The horizontal axis is the number of DEGs associated with each enriched GO term, and the vertical axis is enriched GO terms. BP: Biological Process; CC: Cellular Component; MF: Molecular Function. **(B)** Circular diagram of GO enrichment analysis of DEGs. The outermost layer labels each pathway, while the innermost layer summarizes the P value and enrichment size (gene count). **(C)** KEGG enrichment analysis of DEGs. The horizontal axis is the number of DEGs associated with each enriched KEGG pathway, and the vertical axis is enriched KEGG pathways.

Enrichment analysis was conducted to elucidate the biological functions and pathways linked to the 726 DEGs, thereby revealing the mechanisms through which MDBD influences DR from various perspectives. The DEGs underwent GO enrichment analysis for BP, CC and MF. Findings indicated significant enrichment of DEGs in BP, primarily involving neuron and cell development, encompassing neuron projection regulation, axon development, positive regulation of cell development, axonogenesis, and wound healing. Enrichment in CC included collagen-containing extracellular matrix, neuronal cell body, and endoplasmic reticulum lumen, while MF enrichment comprised growth factor binding, cytokine binding, and voltage-gated monoatomic ion channel activity (**Figures 11A, B**). KEGG enrichment analysis suggested DEG involvement in pathways such as complement and coagulation cascades, pertussis, TGF-beta signaling, focal adhesion, and the PI3K-Akt signaling pathway (**Figure 11C**).

### 3.13 Analysis of DEG clusters and inter-cluster comparisons

**Figure 12:**
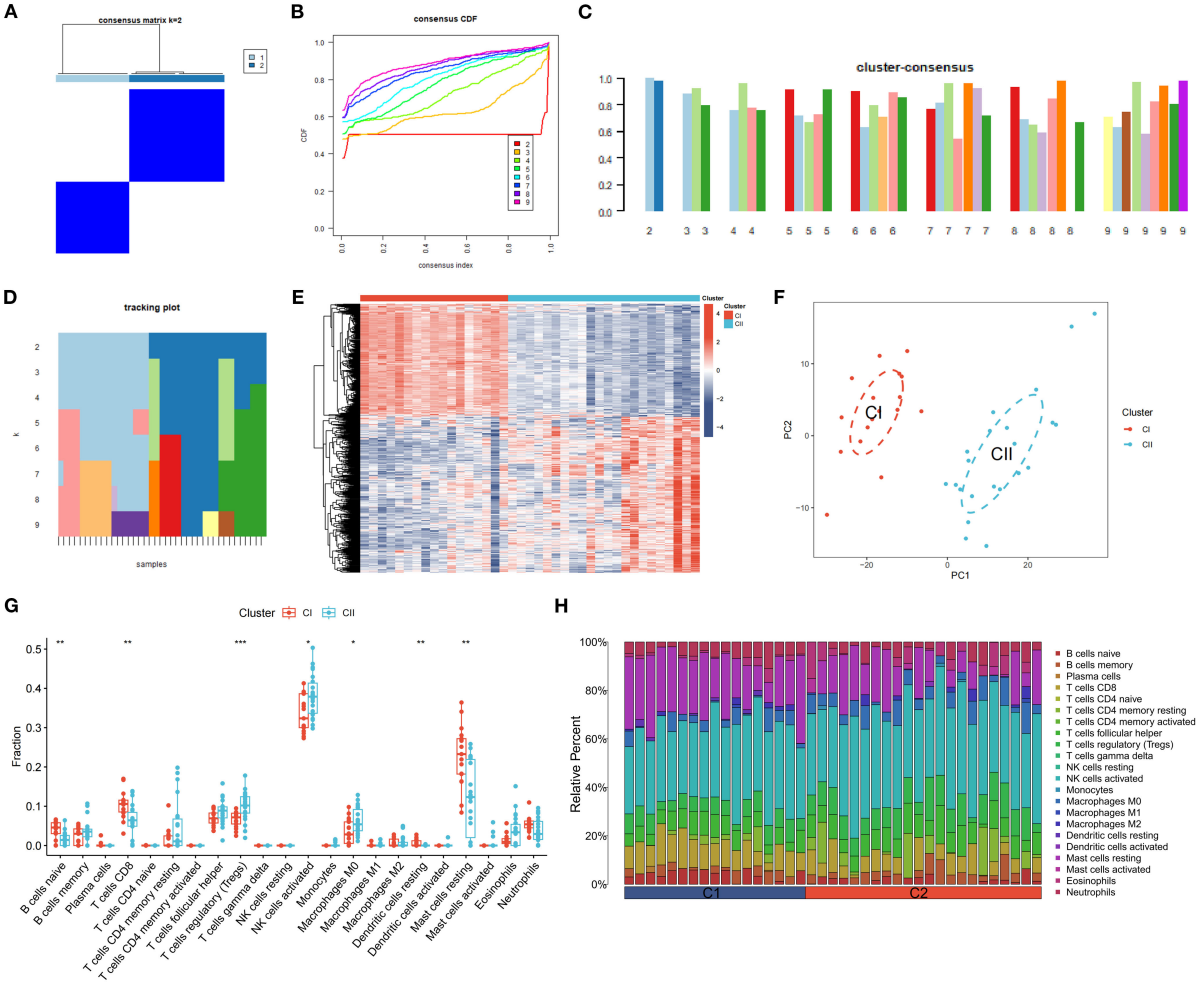
Clustering and Expression Analysis of DEGs. **(A)** A heatmap of the consensus matrix for DEG clustering across samples. **(B)** Cumulative distribution functions from the consensus matrix of DEG clustering. **(C)** Mean consensus scores for various clusters. The horizontal axis shows the number of clusters (k = 2–9), and the vertical axis shows the mean consensus score. Colors represent the respective clusters. **(D)** The tracking of mean consensus scores across different clusters. **(E)** Heatmap of the expression of DEGs between CI and CII. **(F)** PCA scatter plots of DEGs between CI and CII. **(G, H)** The comparison of ssGSEA for immune cells between CI and CII. The horizontal axis is immune cells, and the vertical axis is immune cell fraction in figure 12G. The data are presented as the mean ± SEM. *P<0.05; **P<0.01; ***P<0.001.

K-means clustering, utilizing Euclidean distance with a maximum of nine clusters, was applied to classify DR samples based on the expression of 726 DEGs. This approach resulted in the division of samples into two distinct clusters, achieving precise outcomes (**Figures 12A, B**) and demonstrating high stability (**Figures 12C, D**). A subsequent analysis of DEG expression within these clusters identified 305 DEGs with significantly increased expression levels in Cluster CI and decreased levels in Cluster CII, including CD163L1, PCYT2, SLC45A1, and ELOVL6. Conversely, 421 DEGs showed elevated expression in Cluster CII and reduced levels in Cluster CI, such as LPAR6, CGNL1, SHISA9, and TNFRSF1B (**Figure 12E**). Principal Component Analysis (PCA) revealed a clear distinction between the clusters, with Cluster CI exhibiting a higher density than Cluster CII (**Figure 12F**). ssGSEA results indicated a high expression of naive B cells, CD8+ T cells, resting dendritic cells, and resting mast cells in Cluster CI (*P* < 0.01), whereas regulatory T cells (Tregs), activated NK cells, and M0 macrophages were more prevalent in Cluster CII (*P* < 0.05, *P* < 0.01, *P* < 0.001) (**Figure 12G**). Immune cell infiltration analysis characterized the types and levels of immune cells expressed in each sample from both clusters, visually represented in **Figure 12H**.

### 3.14 Comparative analysis of SDECG scores across clusters and alluvial plot construction

**Figure 13:**
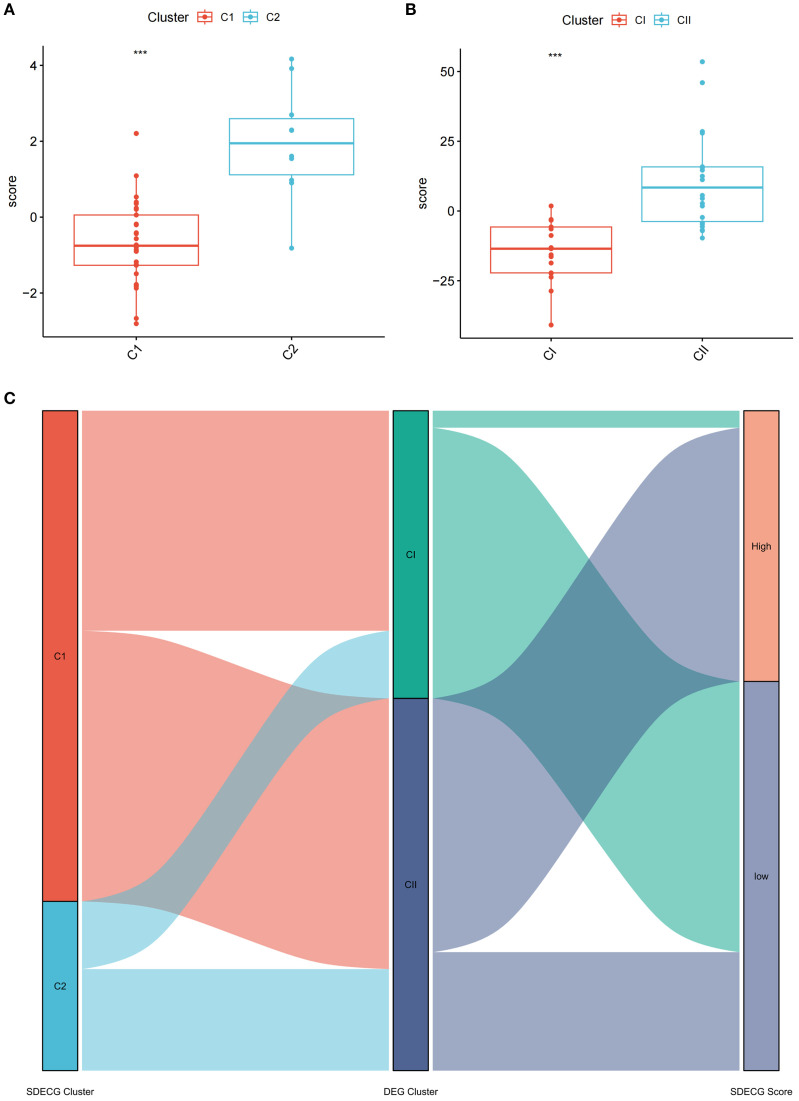
Comparative Analysis of SDECG Scores and Cluster Relationships. **(A)** The scores for SDECG in Clusters C1 and C2. The horizontal axis is sample groups, and the vertical axis is SDECG score of samples. The data are presented as the mean ± SEM. *P<0.05; **P<0.01; ***P<0.001. **(B)** The DEG scores for Clusters CI and CII. The horizontal axis is sample groups, and the vertical axis is DEG score of samples. The data are presented as the mean ± SEM. *P<0.05; **P<0.01; ***P<0.001. **(C)** Alluvial plot of the inter-cluster relationships. The alluvial plot shows the relationships and overall flow between SDECG clusters, DEG clusters, and samples with high and low SDECG scores. Each ribbon represents the flow of samples across SDECG clusters, DEG clusters, and SDECG score groups (High vs Low).

PCA was utilized to evaluate the SDECGs of MDBD in DR treatment, leading to the establishment of a scoring model. Differences in scores among various clusters were assessed to determine significant disparities in SDECGs and to establish the corresponding cluster relationships, ensuring the findings’ reliability. PCA results indicated a statistically significant difference between two SDECG clusters (*P* < 0.001) (**Figure 13A**) and two DEG clusters (*P* < 0.001) (**Figure 13B**), demonstrating that C1 has a higher score than C2, and CI surpasses CII. The alluvial plot suggested that C1 in SDECG clustering aligns with CI in DEG clustering, while C2 corresponds predominantly to CII (**Figure 13C**).

### 3.15 *In vitro* experimental validation of beta-sitosterol effects

**Figure 14:**
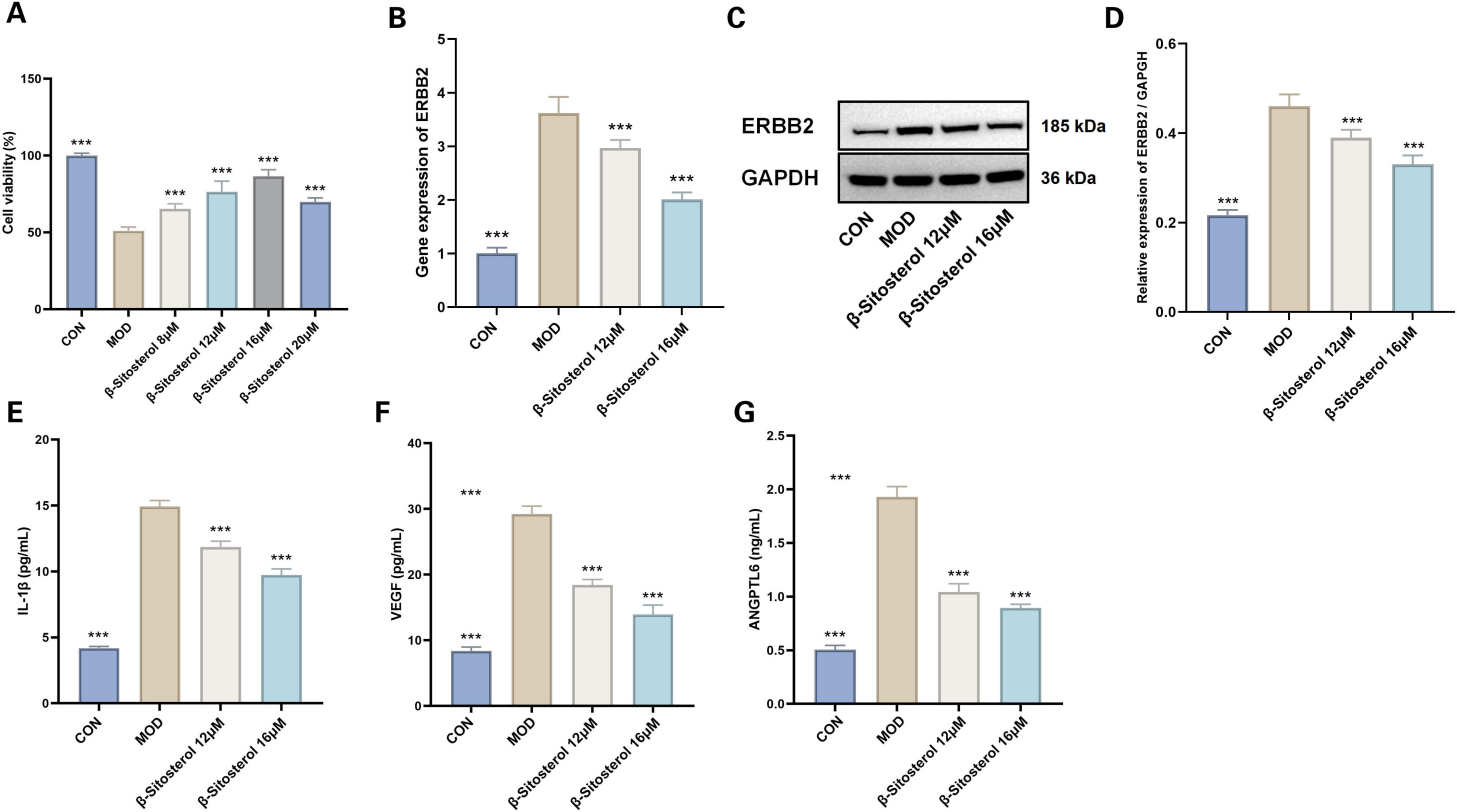
Effects of Beta-sitosterol on Hypoxia-Induced MMCs. **(A)** Cell viability (n=4). The data are presented as the mean ± SEM; ***P < 0.001 vs MOD group. **(B)** RTqPCR results for ERBB2 gene expression (n=6). The data are presented as the mean ± SEM; ***P < 0.001 vs MOD group. **(C, D)** Western blotting results of ERBB2 (n=3). The data are presented as the mean ± SEM; ***P < 0.001 vs MOD group. **(E)** IL-1β levels (n=6). **(F)** VEGF levels(n=6). **(G)** ANGPTL6 levels (n=6). Data presented as mean ± SEM. ***p < 0.001 vs MOD group. CON, Control group; MOD, Model group; β-sitosterol, Beta-sitosterol; VEGF, Vascular endothelial growth factor; ANGPTL6, aAngiopoietin-like protein 6; IL-1β, Interleukin-1 beta.

A cell viability assay demonstrated a significant reduction in viability in the hypoxia-induced model group compared to the control group (*P* < 0.001). However, treatment with beta-sitosterol at concentrations of 8μM, 12μM, 16μM, and 20μM significantly restored cell viability relative to the model group (*P* < 0.001 for all comparisons) (**Figure 14A**). Notably, the 12μM and 16μM beta-sitosterol treatment groups exhibited the most pronounced effects, warranting further investigation.

RT-qPCR analysis revealed a marked upregulation of ERBB2 mRNA expression in the model group compared to the control (*P* < 0.001). Treatment with beta-sitosterol at 12μM and 16μM significantly reversed this upregulation, resulting in a substantial downregulation of ERBB2 mRNA expression (*P* < 0.001 for all comparisons) (**Figure 14B**). Similarly, Western blot analysis showed a significant increase in ERBB2 protein expression in the model group relative to the control, while beta-sitosterol treatment at 12μM and 16μM significantly reduced ERBB2 protein levels (*P* < 0.001 for all comparisons) (**Figures 14C, D**).

ELISA analysis revealed that the hypoxia-induced model group showed a statistically significant elevation in IL-1β, VEGF, and ANGPTL6 levels compared to the control group (*P* < 0.001) (**Figures 14E–G**). Beta-sitosterol treatment at concentrations of 12μM and 16μM effectively mitigated these increases, with the suppressive effects being more pronounced at the higher concentration of 16μM (*P* < 0.001 for all comparisons) (**Figures 14E–G**).

### 3.16 *In vitro* experimental assessment of ERBB2 inhibition effects

**Figure 15:**
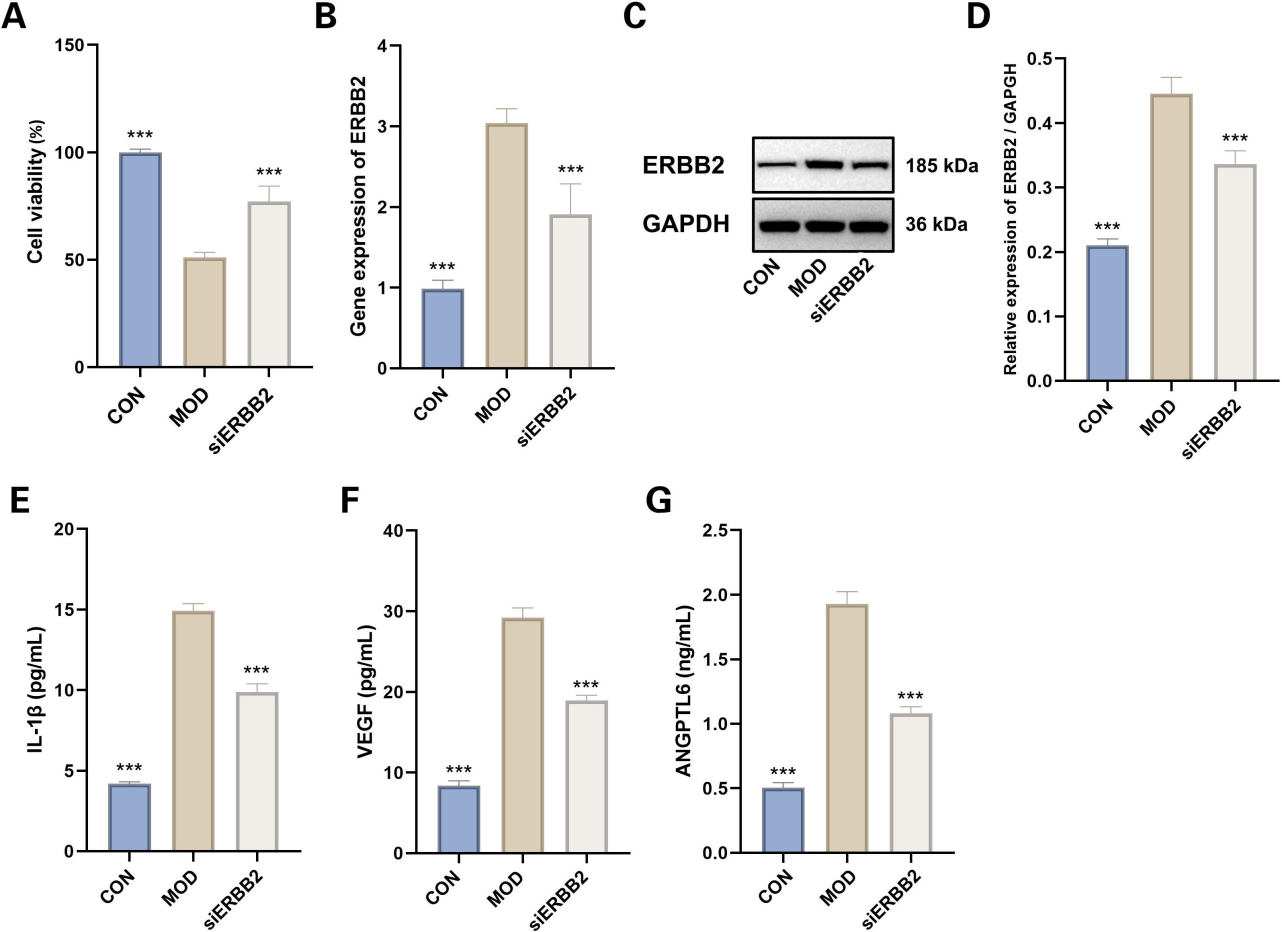
Evaluation of siERBB2 Effects on Hypoxia-Induced MMCs. **(A)** Cell viability (n=4). Data presented as mean ± SEM. ***p < 0.001 vs MOD group. **(B)** RTqPCR results of ERBB2 gene expression (n=6). Data presented as mean ± SEM. ***p < 0.001 vs MOD group. **(C, D)** Western blotting results of ERBB2 (n=3). Data presented as mean ± SEM. ***p < 0.001 vs MOD group. **(E)** IL-1β levels (n=6). **(F)** VEGF levels(n=6). **(G)** ANGPTL6 levels (n=6). Data presented as mean ± SEM. ***p < 0.001 vs MOD group. CON: Control group; MOD: Model group.

Cell viability assays indicated a notable reduction in viability within the model group relative to the control group (*P* < 0.001). Conversely, siERBB2 treatment significantly enhanced cell viability compared to the model group (*P* < 0.001) (**Figure 15A**).

RT-qPCR analysis demonstrated that ERBB2 mRNA expression was substantially upregulated in the model group compared to the control (*P* < 0.001). This upregulation was effectively reversed in the siERBB2 treatment group, where ERBB2 mRNA expression was significantly downregulated compared to the model group (*P* < 0.001) (**Figure 15B**). Western blot analysis similarly confirmed that ERBB2 protein levels were markedly elevated in the model group versus the control group (*P* < 0.001). However, siERBB2 treatment significantly reduced ERBB2 protein expression compared to the model group (*P* < 0.001) (**Figures 15C, D**).

The ELISA results demonstrated a significant increase in IL-1β, VEGF, and ANGPTL6 levels in the hypoxia-induced model group compared to the control group (*P* < 0.001). Furthermore, ERBB2 inhibition notably reduced the elevated levels of IL-1β, VEGF, and ANGPTL6 in the model group, indicating its suppressive effect (*P* < 0.001 for both) (**Figures 15E–G**).

## 4 Discussion

This study identified quercetin, stigmasterol, beta-sitosterol, and kaempferol as the main active components of MDBD. Among the fourteen SDECGs involved in immune regulation, ERBB2 was confirmed as a key risk factor for DR through MR analysis. Beta-sitosterol showed strong binding affinity with multiple feature genes and effectively reduced ERBB2 expression *in vitro*, suppressing IL-1β, VEGF, and ANGPTL6 secretion. These findings highlight the therapeutic potential of MDBD in targeting DR pathogenesis. +Five feature genes (CCND1, ERBB2, INSR, TP53, SERPINE1).

### 4.1 MDBD for DR in TCM

DM, classified within the “Xiao Ke” category in TCM, is characterized by symptoms such as excessive thirst, hunger, urination, and weight loss. Its earliest documentation appears in the Yellow Emperor’s Inner Canon (36). In TCM, DR is referred to as “Xiao Ke Mu Bing,” an ocular complication of “Xiao Ke,” driven by internal heat resulting from yin deficiency. This imbalance disrupts qi and blood flow, leading to stasis and contributing to DR progression (37, 38). MDBD addresses these pathological mechanisms by enhancing qi, nourishing blood, and regulating meridians. Clinical studies have shown that combining MDBD with calcium hydroxybenzenesulfonate reduces inflammatory and angiogenic markers such as high-sensitivity C-reactive protein, tumor necrosis factor-alpha, VEGF, and endothelin-1, effectively ameliorating DR (39, 40).

### 4.2 Bioactive components of MDBD for DR

Through analysis of the “drug-component-target” network, the primary active compounds responsible for MDBD’s therapeutic effects on DR have been identified. Beta-sitosterol, a naturally occurring bioactive phytosterol can be found in Angelica sinensis and Panax notoginseng, is a key component of MDBD. Its chemical structure closely resembles cholesterol from mammalian cells (41), exhibiting antioxidant properties and anti-diabetic activity (42, 43). Beta-sitosterol exhibited high binding affinity with genes implicated in DR pathogenesis. *In vitro* experiments using a hypoxia-induced DR cell model demonstrated that beta-sitosterol significantly reduced IL-1β, VEGF, and ANGPTL6 secretion while improving cell viability, supporting its role in mitigating key pathological processes in DR. Moreover, a research on ADMET (absorption, distribution, metabolism, excretion, and toxicity) profiling on beta-sitosterol (44) verified that beta-sitosterol complies with Lipinski’s Rule of Five, showing high intestinal absorption and effective permeability across the blood-brain barrier. These pharmacokinetic attributes indicate that beta-sitosterol is well-suited for systemic distribution and may serve as a promising candidate for oral administration.

Additionally, compounds such as stigmasterol from Angelica sinensis and Panax notoginseng, quercetin from Astragalus membranaceus, Angelica sinensis and Panax notoginseng, along with jaranol, hederagenin, isorhamnetin, and bifendate from Astragalus membranaceus, collectively contribute to the therapeutic benefits of MDBD. Stigmasterol from displays various pharmacological properties, including anti-diabetic, anti-tumor, anti-inflammatory, and antioxidant effects, and has been shown to inhibit high glucose-induced proliferation and angiogenesis in retinal cells (45, 46). Quercetin reduces inflammation and enhances retinal layer thickness, offering therapeutic benefits in DR (47). Jaranol exhibits antitumor activity against breast and liver cancers and possesses anti-influenza properties (48, 49). Hederagenin demonstrates antitumor and anti-inflammatory effects, and has been shown to attenuate high glucose-induced fibrosis in renal cells (50–52). Isorhamnetin provides cardiovascular protection and boosts insulin secretion from pancreatic β-cells (53, 54). Bifendate is recognized for treating hepatitis, reducing hepatotoxicity, and showing antifibrotic effects (55, 56). Formononetin is reported to ameliorate type 2 diabetes progression and related complications by lowering hyperglycemia and insulin resistance in diabetic rats (57). Calycosin, a phytoestrogen, exhibits potent antioxidant and anti-tumor properties, mitigating kidney injury and cognitive impairments induced by diabetes in animal models (58–60). Kaempferol regulates angiogenesis, apoptosis, metastasis, and inflammation, enhancing diabetes management through modulation of endoplasmic reticulum stress (61, 62). Finally, Ginsenoside Rh2 shows anti-cancer properties against various cancers and improves immune function, effectively reducing elevated fasting blood glucose levels in type 1 diabetes mellitus rats (63, 64). These findings show MDBD’s multi-target therapeutic potential in DR, combining active compounds to address inflammation, oxidative stress, and angiogenesis, thereby providing a comprehensive approach to managing this complex condition.

### 4.3 Core genes targeted by MDBD for DR

The core targets were identified using the PPI network, followed by differential expression analysis to pinpoint SDECGs core genes, which were subsequently validated in human samples. Among the top five SDECGs, ERBB2 emerged as a significant target based on feature importance scores, demonstrating a strong association with increased risk for DR. Beta-sitosterol was found to bind to ERBB2, a receptor tyrosine kinase linked to elevated levels that significantly correlate with a higher incidence of diabetes mellitus. Individuals with high ERBB2 levels exhibit a markedly increased risk of diabetes compared to those with lower levels (65). To validate the relationship between ERBB2 and DR, *in vitro* experiments were conducted using a hypoxia-induced DR cell model. Results revealed significant upregulation of ERBB2 at both mRNA and protein levels. However, treatment with beta-sitosterol (12 μM and 16 μM) and ERBB2 inhibition effectively downregulated these elevated levels, affirming the interaction between ERBB2 and DR. These findings suggest that beta-sitosterol may target the MDBD-related gene ERBB2 to mitigate key pathological processes in DR. Additionally, significant alterations in inflammatory and angiogenic factors associated with DR were observed. The proinflammatory cytokine IL-1β, markedly elevated in the hypoxia-induced model group, was significantly reduced following beta-sitosterol treatment and ERBB2 inhibition, highlighting ERBB2’s role in modulating inflammation. Similarly, angiogenic factors VEGF and ANGPTL6, crucial for pathological neovascularization, were modulated. While their levels were initially increased in the hypoxia-induced model, further downregulation occurred under beta-sitosterol treatment and ERBB2 inhibition, indicating ERBB2’s involvement in angiogenesis regulation in DR.

Apart from ERBB2, the other top four SDECGs identified in our analysis include CCND1, SERPINE1, TP53, and INSR. Each of these genes plays a distinct role in the pathophysiology of DR. While ERBB2 is associated to DR risk and its strong binding affinity with beta-sitosterol, which supports the *in vitro* findings, the inclusion of these additional targets demonstrates their complementary roles in DR pathogenesis and reflects a more comprehensive approach to understanding the DR mechanisms. CCND1, a protein-coding gene activated by insulin, facilitates glycemic normalization via the CCND1-CDK4 pathway (66). SERPINE1 (also known as PAI-1), part of the serine protease inhibitor family, has shown elevated expression in vitreous biopsies and neovascular tissue from patients with PDR (67). TP53, a tumor suppressor protein, exhibited higher expression in blood samples from T2DM patients with DR compared to healthy individuals and T2DM patients without DR (68).

IINSR, an insulin receptor, was identified in a genome-wide association study as strongly correlated with DR in T2DM patients (69). Differential expression analysis in our study further revealed high INSR expression in the DR group, showing its significance in insulin signaling and metabolic regulation. The apparent contradiction in INSR expression across different studies warrants further investigation. Previous studies have indicated that impairments in INSR expression or functionality can lead to insulin resistance and DM (70, 71). To investigate this apparent contradiction, further analysis was conducted. Muscle tissues from DR patients and healthy individuals were analyzed, differing from detection materials in prior studies, such as skeletal muscle (71), peripheral blood lymphocytes (72), and various cell lines, including CEM T-lymphocytes and SW1990 pancreatic cells (73). Additionally, gene-by-environment interactions were found to play a critical role in complex gene regulation. Controlled environmental exposure *in vitro* across different cell types revealed diverse transcriptional responses (74). This variation in INSR expression across cell types may explain its complex regulation within different cellular environments.

By incorporating secondary targets alongside ERBB2, this study adopts a broader perspective on DR pathogenesis. These additional targets provide valuable context about the effect of MDBD, complementing the primary findings without detracting from the significance of ERBB2. Moving forward, a systematic and stepwise evaluation of these targets is essential. Such prioritization should integrate factors including network-level impact, confidence in target–ligand interactions, and functional assessments in retinal endothelial cells under high-glucose conditions.

### 4.4 Immune cell involvement in DR

The ssGSEA analysis revealed significant differences in immune cell expression between normal and DR groups. Positive correlations were observed between SDECGs and immune cells such as eosinophils, macrophages M0, and resting CD4 memory T cells, while negative correlations were found with naive B cells and plasma cells. Among these, macrophages M0 and resting dendritic cells exhibited statistically significant differences. Macrophages M0, precursors to polarized macrophages, can be induced by high glucose levels to polarize into the M1 subtype (75), playing a key role in DR progression (76). Similarly, dendritic cells, which enhance immune responses via antigen presentation, have been identified as a DR risk factor through HLA DR expression in genome-wide association studies (GWAS) (77). Further analysis revealed that macrophages M0 positively correlated with two SDECGs, TP53 and SERPINE1, while resting dendritic cells showed a positive correlation with INSR and a negative correlation with ERBB2. All these correlations demonstrated statistical significance between DR and normal samples. Resting mast cells, on the other hand, were negatively correlated with four SDECGs, TP53, SERPINE1, ERBB2, and CCND1, while positively correlated with INSR, all with statistical significance. However, no significant differences in resting mast cell levels were observed between DR and normal samples, which may be attributed to the distinct and complex regulatory mechanisms between resting mast cells and genetic samples, leading to varied outcomes.

The RF model was utilized to identify feature genes, including the top five for construction, to assess DR onset and the sensitivity and accuracy of MDBD treatment, which showed high sensitivity and accuracy. Enrichment analysis indicated that MDBD mechanisms in DR treatment are linked to immunity and inflammation, aligning with DR pathogenesis and development factors (78). In DR, elevated blood glucose levels lead to mitochondrial dysfunction, inflammation, and increased vascular endothelial growth factor secretion, causing vascular and neuronal apoptosis, and neovascularization (79). The activation of immune cells takes place earlier than neuronal dysfunction and intraretinal microvascular abnormalities (80). Immune cell activation contributes to neuronal dysfunction and intraretinal microvascular abnormalities (81, 82). Elevated inflammatory cytokines such as IFN-γ, TNF, and IL-2, secreted by lymphocytes, have been detected in DR patients (83) and in diabetic rat retinal tissue (84). Additionally, macrophages have been identified in the retinal tissue of Akimba mice, a recognized model of diabetic retinopathy, through single-cell RNA sequencing (85). These results reveal the complex relationship between immune cell activation, genetic factors, and inflammatory processes in the development of DR. The correlations identified between specific immune cells and SDECGs point to the significant role of immune regulation in disease progression. The ability of MDBD to influence these immune-related pathways suggests its therapeutic relevance for DR. Further investigation into the interaction between immune cells and genetic factors will be essential for advancing our understanding of DR and improving treatment strategies.

## 5 Conclusion

This study employed a multidimensional approach, integrating network pharmacology, molecular docking, GEO datasets, and Mendelian randomization analysis, to explore the mechanisms of MDBD in treating DR. Key genes, including INSR, CCND1, ERBB2, TP53, and SERPINE1, were identified as critical targets of MDBD, mediated by core components such as quercetin, stigmasterol, beta-sitosterol, and kaempferol. These interactions alleviate DR by modulating immunity- and inflammation-related pathways. Notably, the inhibition of ERBB2 and the application of beta-sitosterol demonstrated therapeutic efficacy, reducing ERBB2 protein and mRNA levels, as well as key inflammatory and angiogenic factors such as IL-1β, VEGF, and ANGPTL6. These findings provide valuable insights into the molecular mechanisms of MDBD and its potential for future clinical applications, though further experimental validation is required.

## Data Availability

The original contributions presented in the study are included in the article/**Supplementary Material**. Further inquiries can be directed to the corresponding authors.
