# Supplementary material for: Mechanistic insights into modified Danggui Buxue Decoction for diabetic retinopathy via integrative analysis
Source: Front Endocrinol (Lausanne). 2025 Oct 10;16:1648831. doi: 10.3389/fendo.2025.1648831 (PMC12549254; doi:10.3389/fendo.2025.1648831)
Supplement: Supplementary file 1 [file Table1.docx]

**Supplementary Material Table**

| **Table S1 Core components of Modified Danggui Buxue Decoction** | | | | | | |
| --- | --- | --- | --- | --- | --- | --- |
| **Mol ID** | **Molecule Name** | **MW** | **AlogP** | **OB (%) ≥30%** | **DL≥0.18** | **Drug** |
| MOL000358 | beta-sitosterol | 414.79 | 8.08 | 36.91 | 0.75 | Danggui |
| MOL000449 | Stigmasterol | 412.77 | 7.64 | 43.83 | 0.76 | Danggui |
| MOL000211 | Mairin | 456.78 | 6.52 | 55.38 | 0.78 | Huangqi |
| MOL000239 | Jaranol | 314.31 | 2.09 | 50.83 | 0.29 | Huangqi |
| MOL000296 | hederagenin | 414.79 | 8.08 | 36.91 | 0.75 | Huangqi |
| MOL000033 | (3S,8S,9S,10R,13R,14S,17R)-10,13-dimethyl-17-[(2R,5S)-5-propan-2-yloctan-2-yl]-2,3,4,7,8,9,11,12,14,15,16,17-dodecahydro-1H-cyclopenta[a]phenanthren-3-ol | 428.82 | 8.54 | 36.23 | 0.78 | Huangqi |
| MOL000354 | isorhamnetin | 316.28 | 1.76 | 49.6 | 0.31 | Huangqi |
| MOL000371 | 3,9-di-O-methylnissolin | 314.36 | 2.89 | 53.74 | 0.48 | Huangqi |
| MOL000374 | 5'-hydroxyiso-muronulatol-2',5'-di-O-glucoside | 642.67 | -0.95 | 41.72 | 0.69 | Huangqi |
| MOL000378 | 7-O-methylisomucronulatol | 316.38 | 3.38 | 74.69 | 0.3 | Huangqi |
| MOL000379 | 9,10-dimethoxypterocarpan-3-O-β-D-glucoside | 462.49 | 0.74 | 36.74 | 0.92 | Huangqi |
| MOL000380 | (6aR,11aR)-9,10-dimethoxy-6a,11a-dihydro-6H-benzofurano[3,2-c]chromen-3-ol | 300.33 | 2.64 | 64.26 | 0.42 | Huangqi |
| MOL000387 | Bifendate | 418.38 | 2.56 | 31.1 | 0.67 | Huangqi |
| MOL000392 | formononetin | 268.28 | 2.58 | 69.67 | 0.21 | Huangqi |
| MOL000398 | isoflavanone | 316.33 | 2.42 | 109.99 | 0.3 | Huangqi |
| MOL000417 | Calycosin | 284.28 | 2.32 | 47.75 | 0.24 | Huangqi |
| MOL000422 | kaempferol | 286.25 | 1.77 | 41.88 | 0.24 | Huangqi |
| MOL000433 | FA | 441.45 | 0.01 | 68.96 | 0.71 | Huangqi |
| MOL000438 | (3R)-3-(2-hydroxy-3,4-dimethoxyphenyl)chroman-7-ol | 302.35 | 3.13 | 67.67 | 0.26 | Huangqi |
| MOL000439 | isomucronulatol-7,2'-di-O-glucosiole | 626.67 | -0.68 | 49.28 | 0.62 | Huangqi |
| MOL000442 | 1,7-Dihydroxy-3,9-dimethoxy pterocarpene | 314.31 | 3.11 | 39.05 | 0.48 | Huangqi |
| MOL000098 | quercetin | 302.25 | 1.5 | 46.43 | 0.28 | Huangqi |
| MOL001494 | Mandenol | 308.56 | 6.99 | 42 | 0.19 | Sanqi |
| MOL001792 | DFV | 256.27 | 2.57 | 32.76 | 0.18 | Sanqi |
| MOL002879 | Diop | 390.62 | 7.44 | 43.59 | 0.39 | Sanqi |
| MOL000358 | beta-sitosterol | 414.79 | 8.08 | 36.91 | 0.75 | Sanqi |
| MOL000449 | Stigmasterol | 412.77 | 7.64 | 43.83 | 0.76 | Sanqi |
| MOL005344 | ginsenoside rh2 | 622.98 | 4.04 | 36.32 | 0.56 | Sanqi |
| MOL007475 | ginsenoside f2 | 785.14 | 2.3 | 36.43 | 0.25 | Sanqi |
| MOL000098 | quercetin | 302.25 | 1.5 | 46.43 | 0.28 | Sanqi |

| **Table S2 Core targets identified from the PPI network** | | | | | |
| --- | --- | --- | --- | --- | --- |
| **Acronyms of Gene** | **Gene name** | **Degree** | **EcCentricity** | **Closeness** | **Cluster** |
| MAPK8 | Mitogen-activated protein kinase 8 | 43 | 0.5 | 48.5 | 1 |
| CASP9 | Caspase-9 | 43 | 0.5 | 48.5 | 1 |
| IL1A | Interleukin-1 alpha | 51 | 0.5 | 52.5 | 1 |
| IFNG | Interferon gamma | 54 | 1 | 54 | 1 |
| EGFR | Epidermal growth factor receptor | 53 | 0.5 | 53.5 | 1 |
| IL1B | Interleukin-1 beta | 54 | 1 | 54 | 1 |
| CDKN1A | Cyclin dependent kinase inhibitor 1A | 40 | 0.5 | 47 | 1 |
| PPARG | Peroxisome proliferator activated receptor gamma | 52 | 0.5 | 53 | 1 |
| PPARA | Peroxisome proliferator-activated receptor alpha | 36 | 0.5 | 45 | 1 |
| CASP3 | Caspase-3 | 54 | 1 | 54 | 1 |
| NFKBIA | Nuclear factor of kappa light polypeptide gene enhancer in B-cells inhibitor alpha | 52 | 0.5 | 53 | 1 |
| NFE2L2 | Nuclear factor erythroid 2-related factor 2 | 43 | 0.5 | 48.5 | 1 |
| GSK3B | Glycogen synthase kinase 3 beta | 43 | 0.5 | 48.5 | 1 |
| FOS | Fos proto-oncogene | 52 | 0.5 | 53 | 1 |
| IL4 | Interleukin 4 | 46 | 0.5 | 50 | 1 |
| HSP90AA1 | Heat shock protein 90 alpha family class A member 1 | 46 | 0.5 | 50 | 1 |
| IL10 | Interleukin-10 | 50 | 0.5 | 52 | 1 |
| CASP8 | Caspase 8 | 45 | 0.5 | 49.5 | 1 |
| ICAM1 | Intercellular adhesion molecule 1 | 53 | 0.5 | 53.5 | 1 |
| CAV1 | Caveolin-1 | 37 | 0.5 | 45.5 | 1 |
| STAT1 | Signal transducer and activator of transcription 1 | 48 | 0.5 | 51 | 1 |
| PARP1 | Poly(ADP-ribose) polymerase 1 | 40 | 0.5 | 47 | 1 |
| IKBKB | Inhibitor of nuclear factor kappa B kinase subunit beta | 41 | 0.5 | 47.5 | 1 |
| CCND1 | Cyclin D1 | 49 | 0.5 | 51.5 | 1 |
| PTGS2 | Prostaglandin-endoperoxide synthase 2 | 54 | 1 | 54 | 1 |
| MAPK1 | Mitogen-activated protein kinase 1 | 38 | 0.5 | 46 | 1 |
| CASP1 | Caspase-1 | 41 | 0.5 | 47.5 | 1 |
| MMP9 | Matrix Metallopeptidase 9 | 54 | 1 | 54 | 1 |
| TNF | Tumor necrosis factor | 54 | 1 | 54 | 1 |
| CHUK | Component of inhibitor of nuclear factor kappa B kinase complex | 37 | 0.5 | 45.5 | 1 |
| VCAM1 | Vascular cell adhesion protein 1 | 44 | 0.5 | 49 | 1 |
| BCL2L1 | BCL2 like 1 | 48 | 0.5 | 51 | 1 |
| MMP2 | Matrix metallopeptidase 2 | 48 | 0.5 | 51 | 1 |
| MMP3 | Matrix metallopeptidase 3 | 37 | 0.5 | 45.5 | 1 |
| BCL2 | B-cell leukemia/lymphoma 2 protein | 54 | 1 | 54 | 1 |
| SERPINE1 | Serpin family E member 1 | 41 | 0.5 | 47.5 | 1 |
| PLAU | Plasminogen activator, urokinase | 36 | 0.5 | 45 | 1 |
| MPO | Myeloperoxidase | 31 | 0.5 | 42.5 | 1 |
| IL6 | Interleukin-6 | 54 | 1 | 54 | 1 |
| CXCL8 | C-X-C motif chemokine ligand 8 | 52 | 0.5 | 53 | 1 |
| CCL2 | C-C motif chemokine ligand 2 | 52 | 0.5 | 53 | 1 |
| CXCL10 | C-X-C motif chemokine ligand 10 | 36 | 0.5 | 45 | 1 |
| ESR1 | Estrogen receptor 1 | 52 | 0.5 | 53 | 1 |
| HIF1A | Hypoxia inducible factor 1 subunit alpha | 54 | 1 | 54 | 1 |
| AKT1 | AKT serine/threonine kinase 1 | 54 | 1 | 54 | 1 |
| MAPK14 | Mitogen-activated protein kinase 14 | 44 | 0.5 | 49 | 1 |
| KDR | Kinase insert domain receptor | 39 | 0.5 | 46.5 | 1 |
| MMP1 | Matrix metallopeptidase 1 | 36 | 0.5 | 45 | 1 |
| MYC | MYC proto-oncogene, bHLH transcription factor | 52 | 0.5 | 53 | 1 |
| EGF | Epidermal growth factor | 49 | 0.5 | 51.5 | 1 |
| HSPA5 | Heat shock protein family A (Hsp70) member 5 | 37 | 0.5 | 45.5 | 1 |
| SPP1 | Secreted phosphoprotein 1 | 39 | 0.5 | 46.5 | 1 |
| TP53 | Tumor protein p53 | 54 | 1 | 54 | 1 |
| HMOX1 | Heme oxygenase 1 | 51 | 0.5 | 52.5 | 1 |
| ERBB2 | Erb-b2 receptor tyrosine kinase 2 | 47 | 0.5 | 50.5 | 1 |
| SOD1 | Superoxide dismutase 1 | 4 | 0.33333 | 10 | 2 |
| NOS3 | Nitric oxide synthase 3 | 4 | 0.25 | 8.91667 | 2 |
| CD40LG | CD40 ligand | 3 | 0.25 | 8.08333 | 2 |
| AR | Androgen receptor | 9 | 0.25 | 12.5 | 2 |
| SELE | Selectin E | 4 | 0.2 | 7.91667 | 2 |
| IGFBP3 | Insulin like growth factor binding protein 3 | 3 | 0.2 | 8.65 | 2 |
| HSPB1 | Heat shock protein family B (small) member 1 | 4 | 0.33333 | 10.5 | 2 |
| PLAT | Plasminogen activator, tissue type | 3 | 0.2 | 7.41667 | 2 |
| CCNA2 | Cyclin A2 | 4 | 0.25 | 10 | 2 |
| CDK2 | Cyclin dependent kinase 2 | 8 | 0.33333 | 12.66667 | 2 |
| IGF2 | Insulin like growth factor 2 | 4 | 0.2 | 9.15 | 2 |
| RUNX2 | RUNX family transcription factor 2 | 4 | 0.2 | 9.31667 | 2 |
| E2F1 | E2F transcription factor 1 | 4 | 0.25 | 10 | 2 |
| IRF1 | Interferon regulatory factor 1 | 3 | 0.33333 | 9.66667 | 2 |
| CDK1 | Cyclin dependent kinase 1 | 7 | 0.25 | 11.5 | 2 |
| MET | MET proto-oncogene, receptor tyrosine | 2 | 0.2 | 8.15 | 2 |
| NOS2 | Nitric oxide synthase 2 | 4 | 0.25 | 8.91667 | 2 |
| COL1A1 | Collagen type I alpha 1 chain | 4 | 0.2 | 9.31667 | 2 |
| BAX | BCL2 Associated X, Apoptosis Regulator | 2 | 0.25 | 8.5 | 2 |

|  | **Table S3 SNPs information for the 4 feature genes** | | | | | | | | | | |  |
| --- | --- | --- | --- | --- | --- | --- | --- | --- | --- | --- | --- | --- |
| **Gene** | **SNP** | **A1** | **A2** | **Chr** | **Sample size** | **beta** | **se** | **p** | **eaf** | **pos.exposure** | **R2** | **F** |
| CCND1 | rs7589501 | G | A | 2 | 31315 | 0.0758054 | 0.0118866 | 1.80E-10 | 0.49719 | 60557705 | 0.001311221 | 40.66838722 |
|  | rs7570971 | A | C | 2 | 27240 | -0.0738292 | 0.0119707 | 6.93E-10 | 0.441154 | 135837906 | 0.001523039 | 38.03491047 |
|  | rs34794906 | C | T | 6 | 27835 | -0.0808813 | 0.0128454 | 3.04E-10 | 0.310471 | 31237858 | 0.001453323 | 39.64323558 |
|  | rs10098310 | A | G | 8 | 31046 | 0.0745761 | 0.0120234 | 5.55E-10 | 0.575007 | 130613614 | 0.001227041 | 38.46954118 |
|  | rs597808 | G | A | 12 | 24937 | 0.105669 | 0.0118837 | 6.01E-19 | 0.52377 | 111973358 | 0.002832492 | 79.06069534 |
|  | rs8067378 | G | A | 17 | 30977 | -0.0897236 | 0.0118797 | 4.27E-14 | 0.497388 | 38051348 | 0.001834001 | 57.03933529 |
|  | rs6074022 | T | C | 20 | 31046 | -0.103624 | 0.0136587 | 3.28E-14 | 0.746768 | 44740196 | 0.001850512 | 57.55379878 |
| ERBB2 | rs10489481 | A | G | 1 | 31355 | 0.120783 | 0.0127787 | 3.33E-21 | 0.314428 | 185391167 | 0.002841161 | 89.33272319 |
|  | rs4848370 | T | C | 2 | 31355 | 0.0847329 | 0.0133967 | 2.54E-10 | 0.269386 | 111811665 | 0.00127423 | 40.00191421 |
|  | rs4256159 | T | C | 3 | 31470 | 0.104451 | 0.0173838 | 1.87E-09 | 0.135199 | 18767404 | 0.001145885 | 36.10008042 |
|  | rs7911264 | C | T | 10 | 31470 | -0.06728 | 0.0118955 | 1.55E-08 | 0.515223 | 94436851 | 0.001015473 | 31.9874015 |
|  | rs2617170 | C | T | 12 | 31086 | -0.0900777 | 0.0126158 | 9.33E-13 | 0.668027 | 10560957 | 0.001637301 | 50.97732517 |
|  | rs17761864 | A | C | 17 | 31355 | 0.085127 | 0.0127002 | 2.05E-11 | 0.323707 | 2171637 | 0.00143082 | 44.92476706 |
|  | rs903506 | A | G | 17 | 31268 | -0.102067 | 0.0125251 | 3.68E-16 | 0.658916 | 37879762 | 0.002119273 | 66.40190153 |
|  | rs55908509 | A | G | 19 | 22490 | -0.160209 | 0.0126013 | 4.96E-37 | 0.3284 | 16442019 | 0.007135814 | 161.6234952 |
| INSR | rs6510960 | G | A | 19 | 31684 | 0.143378 | 0.0193011 | 1.10E-13 | 0.105942 | 7203732 | 0.001738622 | 55.17895857 |
|  | rs12642 | A | C | 19 | 31684 | -0.113862 | 0.0171515 | 3.17E-11 | 0.139502 | 7112645 | 0.001389024 | 44.06826838 |
|  | rs4640294 | A | G | 19 | 28457 | -0.123037 | 0.0138826 | 7.82E-19 | 0.759259 | 7297710 | 0.002752603 | 78.54152356 |
| SERPINE1 | rs1354034 | C | T | 3 | 31684 | 0.0910241 | 0.0122264 | 9.69E-14 | 0.618137 | 56849749 | 0.001746291 | 55.42276287 |
|  | rs2227667 | G | A | 7 | 31355 | -0.135254 | 0.0144809 | 9.60E-21 | 0.213451 | 100774749 | 0.00277457 | 87.23312365 |
|  | rs11553699 | G | A | 12 | 30031 | 0.106351 | 0.0190289 | 2.29E-08 | 0.10963 | 122216910 | 0.001039045 | 31.23394664 |
|  | rs12589699 | G | A | 14 | 31684 | -0.108864 | 0.0177967 | 9.52E-10 | 0.127908 | 69212885 | 0.001179606 | 37.41641734 |

|  | **Table S4 MR Analysis of feature genes and DR** | | | |  |
| --- | --- | --- | --- | --- | --- |
| **Gene** | **ID number** | **MR Methods** | **N SNPs** | **OR (95%CI)** | P value |
| CCND1 | eqtl-a-ENSG00000119866 | MR Egger | 7 | 0.084(0.006,1.187) | 0.126177366 |
|  |  | Weighted median | 7 | 1.177(0.704,1.966) | 0.534402937 |
|  |  | Inverse variance weighted | 7 | 1.254(0.841,1.870) | 0.267629905 |
|  |  | Simple mode | 7 | 1.155(0.449,2.972) | 0.774989381 |
|  |  | Weighted mode | 7 | 0.995(0.476,2.081) | 0.990391163 |
| ERBB2 | eqtl-a-ENSG00000141736 | MR Egger | 8 | 0.872(0.111,6.836) | 0.90048654 |
|  |  | Weighted median | 8 | 1.860(1.247,2.774) | 0.002343871 |
|  |  | Inverse variance weighted | 8 | 1.704(1.016,2.856) | 0.043247023 |
|  |  | Simple mode | 8 | 1.902(1.104,3.279) | 0.05373763 |
|  |  | Weighted mode | 8 | 1.888(1.192,2.990) | 0.030238564 |
| INSR | eqtl-a-ENSG00000171105 | MR Egger | 3 | 3.946(0.009,1803.382) | 0.736491809 |
|  |  | Weighted median | 3 | 0.840(0.486,1.450) | 0.53073397 |
|  |  | Inverse variance weighted | 3 | 0.907(0.549,1.498) | 0.70310272 |
|  |  | Simple mode | 3 | 0.840(0.417,1.690) | 0.672676008 |
|  |  | Weighted mode | 3 | 0.840(0.415,1.699) | 0.674919705 |
| SERPINE1 | eqtl-a-ENSG00000106366 | MR Egger | 4 | 5.401(0.277,105.270) | 0.381546311 |
|  |  | Weighted median | 4 | 1.035(0.573,1.870) | 0.909882777 |
|  |  | Inverse variance weighted | 4 | 1.038(0.641,1.682) | 0.878283744 |
|  |  | Simple mode | 4 | 0.716(0.306,1.675) | 0.497585732 |
|  |  | Weighted mode | 4 | 1.323(0.655,2.673) | 0.492418847 |

|  | **Table S5 Heterogeneity and Pleiotropy of MR Analysis results** | | | | | |  |
| --- | --- | --- | --- | --- | --- | --- | --- |
|  | **Heterogeneity** | | | | **Pleiotropy** | | |
| **Gene** | **MR Egger** | | **IVW** | | **MR Egger** | | |
|  | **Cochran’s Q** | **P-value** | **Cochran’s Q** | **P-value** | **Egger intercept** | **P-value** | |
| CCND1 | 2.12 | 0.821 | 6.291 | 0.391 | 0.239 | 0.099 | |
| ERBB2 | 19.48 | 0.003 | 20.897 | 0.004 | 0.073 | 0.533 | |
| INSR | 0.045 | 0.832 | 0.268 | 0.875 | -0.184 | 0.719 | |
| SERPINE1 | 0.782 | 0.676 | 1.998 | 0.573 | -0.185 | 0.385 | |

| **Table S6 Molecular Docking Result** | | | | |
| --- | --- | --- | --- | --- |
| **CID**  **Compound**    **Protein** | **Kaempferol** | **Stigmasterol** | **Beta-sitosterol** | **Quercetin** |
|  | **MOL000422** | **MOL000449** | **MOL000358** | **MOL000098** |
| **CCND1** | -7.6 | -6.8 | -8.3 | -7.4 |
| **ERBB2** | -7.4 | -7.9 | -8.5 | -7.5 |
| **INSR** | -7.4 | -8 | -8.6 | -6.8 |
| **SERPINE1** | -7.8 | -8.6 | -9.3 | -7.7 |
| **TP53** | -6.9 | -6.8 | -7.8 | -7.1 |
