# Supplementary figures and images for: Mechanistic insights into modified Danggui Buxue Decoction for diabetic retinopathy via integrative analysis
Source: Front Endocrinol (Lausanne). 2025 Oct 10;16:1648831. doi: 10.3389/fendo.2025.1648831 (PMC12549254; doi:10.3389/fendo.2025.1648831)

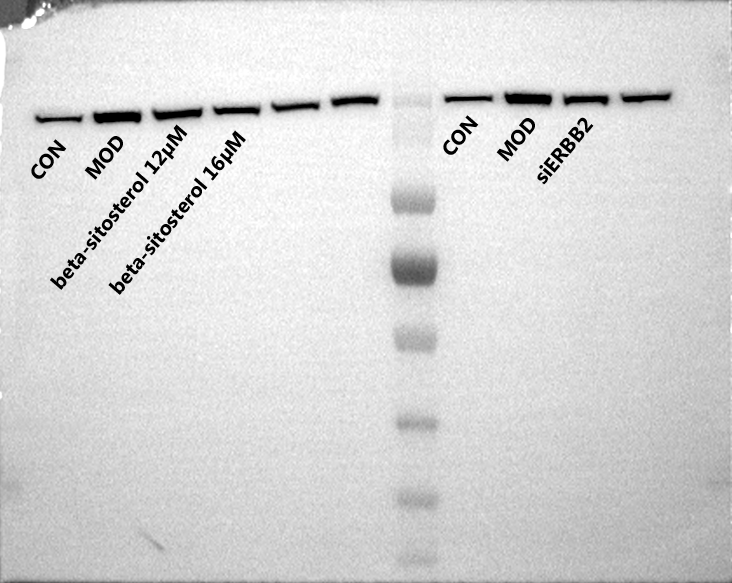

Supplement: Supplementary file 2 [file Image1.tif]

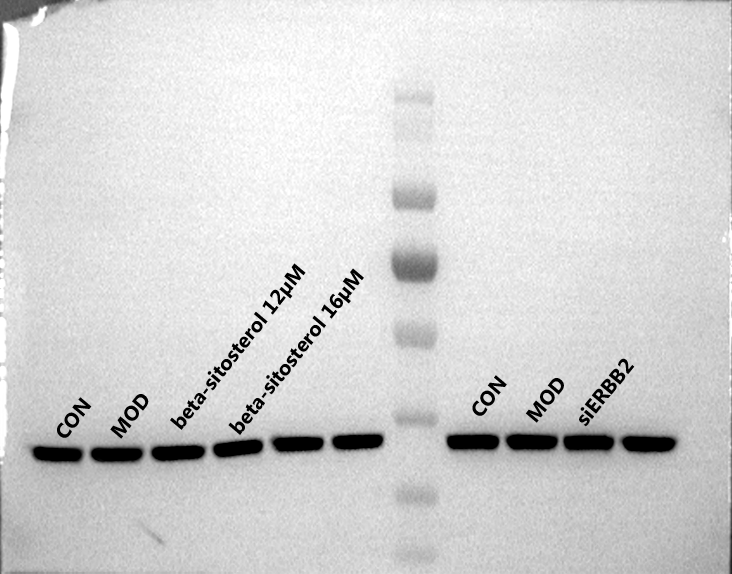

Supplement: Supplementary file 3 [file Image2.tif]
